# Influence of the mating design on the additive genetic variance in plant breeding populations

**DOI:** 10.1007/s00122-023-04447-2

**Published:** 2023-10-31

**Authors:** Tobias Lanzl, Albrecht E. Melchinger, Chris-Carolin Schön

**Affiliations:** 1 Plant Breeding, TUM School of Life Sciences, Technical University of Munich, 85354 Freising, Germany; 2 Institute of Plant Breeding, Seed Science and Population Genetics, University of Hohenheim, 70599 Stuttgart, Germany

## Abstract

**Key message:**

Mating designs determine the realized additive genetic variance in a population sample. Deflated or inflated variances can lead to reduced or overly optimistic assessment of future selection gains.

**Abstract:**

The additive genetic variance ${V}_{A}$ inherent to a breeding population is a major determinant of short- and long-term genetic gain. When estimated from experimental data, it is not only the additive variances at individual loci (QTL) but also covariances between QTL pairs that contribute to estimates of ${V}_{A}$. Thus, estimates of ${V}_{A}$ depend on the genetic structure of the data source and vary between population samples. Here, we provide a theoretical framework for calculating the expectation and variance of ${V}_{A}$ from genotypic data of a given population sample. In addition, we simulated breeding populations derived from different numbers of parents (*P* = 2, 4, 8, 16) and crossed according to three different mating designs (disjoint, factorial and half-diallel crosses). We calculated the variance of ${V}_{A}$ and of the parameter *b* reflecting the covariance component in ${V}_{A},$ standardized by the genic variance. Our results show that mating designs resulting in large biparental families derived from few disjoint crosses carry a high risk of generating progenies exhibiting strong covariances between QTL pairs on different chromosomes. We discuss the consequences of the resulting deflated or inflated ${V}_{A}$ estimates for phenotypic and genome-based selection as well as for applying the usefulness criterion in selection. We show that already one round of recombination can effectively break negative and positive covariances between QTL pairs induced by the mating design. We suggest to obtain reliable estimates of ${V}_{A}$ and its components in a population sample by applying statistical methods differing in their treatment of QTL covariances.

**Supplementary Information:**

The online version contains supplementary material available at 10.1007/s00122-023-04447-2.

## Introduction

The first step in a plant breeding scheme is to generate new variation by crossing promising genotypes to produce the population on which selection is executed. Conditional on the dimension of the breeding program the breeder decides how many and which parents to cross and how many progenies to generate in total and per cross. Thus, a breeding population can range from a bi-parental cross to a complex crossing scheme tracing back to many parents. In the literature, we find large variation across breeding programs with respect to these decisions even for the same crop. For example, in maize, Lian et al. (2015) described a commercial hybrid breeding program in which on average 156 progenies per cross were tested for a large number of biparental crosses at different levels of inbreeding. On the other hand, Auinger et al. (2021) reported on average four progenies per cross, all fully homozygous and derived from bi- or multiparental crosses. The genetic structure of the resulting populations has received little attention in phenotypic selection, but when selection is based on methods that require reliable estimates of the additive genetic variance (${V}_{A}$), such as the usefulness of crosses or genomic and multi-trait selection, population structure and its effect on ${V}_{A}$ cannot be ignored.

${V}_{A}$ quantifies the observable genetic properties of a population (Falconer and Mackay 1996) and when estimated from experimental data it strongly depends on the genetic structure of the data source. In the absence of epistasis, ${V}_{A}$ is composed of the genic variance ${V}_{g}$, which is the sum of the variances of additive effects at individual quantitative trait loci (QTL) and the disequilibrium component $C$, which is twice the sum of the covariances of additive effects between QTL pairs (Lynch and Walsh 1998). Even when sampled from the same population, estimates of the two components can vary considerably among samples, ${V}_{g}$ due to differences in allele frequency spectra and *C* due to variation in gametic phase disequilibrium (GPD) (Falconer and Mackay 1996; Lynch and Walsh 1998).

Avery and Hill (1977) derived theoretical results on the variance of ${V}_{A}$ among replicated small populations sampled from a base population. They concluded that individual samples did not yield accurate predictions of the variance in the base population mainly due to large variation in GPD among samples. Lehermeier et al. (2017a) compared statistical methods for genomic variance estimation in an Arabidopsis data set. They demonstrated that covariances between QTL pairs generated by population structure can lead to over- or underestimation of ${V}_{A}$ calculated based on genomic data unless the estimation method accounted for them.

For a given trait, the contribution of QTL covariances to ${V}_{A}$ depends on the QTL substitution effects and the GPD in the population. We can find both, positive and negative QTL covariances in breeding populations. As is known from theory, negative QTL covariances are expected when traits are under strong directional selection (Bulmer 1971). When introgressing non-adapted material into elite germplasm we might find positive covariances for traits under diversifying selection such as flowering time (Lehermeier et al. 2017a). Recombination will reduce GPD and the covariance component $C$ when intermating the population under study. For real life data, it is therefore not possible to predict the magnitude and sign of the covariance component $C$ and its relative contribution to ${V}_{A}$. Nevertheless, breeders are interested how the design of a breeding program affects the covariance component $C$ relative to the variation in the genic variance. Large variation in $C$ and consequently in ${V}_{A}$ creates uncertainty when estimating quantitative genetic parameters such as trait correlations and when predicting temporal changes of ${V}_{A}$ over breeding cycles (Lara et al. 2022; Allier et al. 2019b). Using theory and simulation results, we investigated for a given trait and population sample the magnitude and sign of the covariance component $C$ and its relative contribution to ${V}_{A}$ conditional on the ancestral population, the crossing scheme and the number of parents sampled from the ancestral population.

In plant breeding, it has been notoriously difficult to obtain meaningful estimates of ${V}_{A}$ from populations generated solely for the purpose of selection (Bernardo 2020). Instead, for quantitative genetic studies, mating designs of different complexity have been devised to estimate ${V}_{A}$ and differentiate between its additive, dominance and epistatic components (Hallauer et al. 2010). Bernardo (2020) defines a mating design as a systematic method for the development of progeny. Using three different mating designs commonly employed in plant breeding, we generated in silico populations varying in allele spectra and levels of GPD. We based our simulations on genotypic data from two published maize breeding experiments to warrant realistic GPD patterns (Mayer et al. 2020; Schrag et al. 2019). The three designs were chosen to resemble breeding populations of different genetic structure sampled from a base population. Making certain assumptions about the distribution of the QTL substitution effects, the variance of the covariance component $C$ and consequently of ${V}_{A}$ can be inferred for each design allowing us to quantify uncertainty of variance estimation in breeding populations of different origin and structure.

We present the theoretical framework for calculating the expectation and variance of the disequilibrium component $C$ in ${V}_{A}$ conditional on the sampled population. Using this framework in combination with simulations we investigated the covariances between QTL pairs on the same and on different chromosomes in different mating designs. We generated populations with (1) parents from two different ancestral populations, one consisting of elite breeding lines, the other of doubled haploid (DH) lines derived from a landrace, (2) different numbers of parents sampled from the ancestral population, (3) different population sizes in subsequent intermating generations, (4) different numbers of additional intermating generations, and (5) quantitative traits governed by different numbers of QTL. Our results are applicable to many populations encountered in plant breeding and can assist breeders in the choice of the design, number and size of the intermating generations for generating new base populations.

## Material and methods

In this study we assume absence of dominance and epistasis and concentrate on fully homozygous material, like e.g. DH lines.

Let ${\mathbf{X}}=\left({x}_{ni}\right)$ denote a $N\times L$ matrix of genotypic scores, where $N$ is the number of genotypes, $L$ is the number of QTL affecting the trait, with ${x}_{ni}=2$, if the $n$th individual is homozygous for the reference allele at the $i$th QTL, and ${x}_{ni}=0$ otherwise. Let $\bf 1$ denote a $N \times 1$ vector of ones and $$\mathbf{p}=\mathbf{1}^{{\varvec{T}}}\mathbf{X}\frac{1}{2N}=\left(\begin{array}{cc}\begin{array}{cc}{p}_{1},& {p}_{2},\end{array}\dots & \begin{array}{cc},{p}_{i},\dots & {p}_{L}\end{array}\end{array}\right)$$ the $1 \times L$ vector of allele frequencies in the population sample considered, where ${p}_{i}$ is the frequency of the reference allele at the $i$th QTL.

Centering with the allele frequencies, we get

1 $${\mathbf{Z}} = {\mathbf{X}} - 2 {\mathbf{1p}}$$

and the $L \times L$ variance–covariance matrix ${\mathbf{D}}$ of genotypic scores

2 $${\mathbf{D}} = {\mathbf{Z}}^{{\varvec{T}}} {\mathbf{Z}}\frac{1}{N}$$

The diagonal elements $\mathbf{D}$ are ${d}_{ii}$= $4{p}_{i}\left(1-{p}_{i}\right)$ and the off-diagonal elements are ${d}_{ij}=4({f}_{ij}-{p}_{i}{p}_{j}) ,$ where the term in parentheses refers to the GPD between locus $i$ and $j$ as defined by Falconer and Mackay (1996), i.e., ${f}_{ij}$ is the gamete frequency and ${p}_{i}$ and ${p}_{j}$ are the allele frequencies of the reference allele at these loci.

We define the following three matrices $\mathbf{V},\mathbf{W},\mathbf{B}$ composed of elements of $\mathbf{D}$

$${\mathbf{V}} = diag\left( {\mathbf{D}} \right) = \left( {v_{{ij}} } \right){\text{ with}}\left\{ {\begin{array}{*{20}c} {v_{{ij}} = d_{{ii}} } & {{\text{if }}i = j} \\ {v_{{ij}} = 0} & {{\text{elsewhere }}} \\ \end{array} } \right\}$$

$${\mathbf{W}} = \left( {w_{{ij}} } \right){\text{ with}}\left\{ {\begin{array}{*{20}c} {w_{{ij}} = d_{{ij}} } & {{\text{if }}\left( {i,j} \right) \in W} \\ {w_{{ij}} = 0} & {{\text{elsewhere }}} \\ \end{array} } \right\}$$

$${\mathbf{B}} = \left( {b_{{ij}} } \right){\text{ with}}\left\{ {\begin{array}{*{20}c} {b_{{ij}} = d_{{ij}} } & {{\text{if }}\left( {i,j} \right) \in B} \\ {b_{{ij}} = 0} & {{\text{elsewhere}}} \\ \end{array} } \right\}$$

where $W$ and $B$ are sets of QTL pairs $\left(i,j\right)$ (with $i\ne j$ and counting $\left(i,j\right)$ and $\left(j,i\right)$ as different pairs) located on the same chromosome or on different chromosomes, respectively.

Let $$\mathbf{a}=\left(\begin{array}{c}\begin{array}{c}{a}_{1}\\ {a}_{2}\end{array}\\ \begin{array}{c}\vdots \\ {a}_{L}\end{array}\end{array}\right)$$ be the vector of fixed effects of the reference allele at the QTL for a given trait.

The additive genetic variance ${V}_{A}$ of the population sample is obtained as the sum of the components ${V}_{g}$ (the sum of the variances of additive effects at individual QTL) and $C$ (the sum of the covariances of additive effects between all QTL pairs), which can be partitioned into ${C}_{w}$ within chromosomes (the sum of the covariances of additive effects of QTL pairs located on the same chromosome) and ${C}_{b}$ between chromosomes (the sum of the covariances of additive effects of QTL pairs located on different chromosomes) (Lara et al. 2022; Lynch and Walsh 1998)

3 $$V_{A} = V_{g} + C = V_{g} + C_{w} + C_{b}$$

These terms can be expressed by quadratic forms as

4 $$V_{A} = {\mathbf{a}}^{T} {\mathbf{Da}}, V_{g} = {\mathbf{a}}^{{\varvec{T}}} {\mathbf{Va}}, C_{w} = {\mathbf{a}}^{{\varvec{T}}} {\mathbf{Wa}}, C_{b} = {\mathbf{a}}^{{\varvec{T}}} {\mathbf{Ba}}$$

and we obtain

5 $${\mathbf{a}}^{{\varvec{T}}} {\mathbf{Da}} = {\mathbf{a}}^{{\varvec{T}}} {\mathbf{Va}} + {\mathbf{a}}^{{\varvec{T}}} {\mathbf{Wa}} + {\mathbf{a}}^{{\varvec{T}}} {\mathbf{Ba}}$$

Assuming the vector $\mathbf{a}$ of fixed QTL effects was sampled for each trait from a multivariate normal distribution with $\mathbf{a}\sim N\left(0,\mathbf{I}\right)$, we get conditional on the matrix $\mathbf{X}$ the following complete formulas for the expectations and variances for the terms in Eq. 5 across different traits (for details see Eqs. A1, A2 in Appendix A)

6 $$E\left[ {V_{g} |{\mathbf{X}}} \right] = trace\left( {\mathbf{V}} \right) = \mathop \sum \limits_{i=1}^{L} d_{ii} ,E\left[ {C_{w} |{\mathbf{X}}} \right] = 0,{ }E\left[ {C_{b} |{\mathbf{X}}} \right] = 0$$

and consequently

7 $$E\left[ {V_{A} |{\mathbf{X}}} \right] = trace\left( {\mathbf{D}} \right) = trace\left( {\mathbf{V}} \right) = \mathop \sum \limits_{i = 1}^{L} d_{ii}$$

8 $${\text{var}} \left[ {V_{g} |{\mathbf{X}}} \right] = 2trace\left( {{\mathbf{V}}^{2} } \right) = 2\mathop \sum \limits_{i = 1}^{L} d_{ii}^{2}$$

9 $$ {\text{var}} \left[ {C_{w} |{\mathbf{X}}} \right] = 2trace\left( {{\mathbf{W}}^{2} } \right) = 2\mathop \sum \limits_{{\left( {i,j} \right) \in W}} d_{ij}^{2}$$

10 $${\text{var}} \left[ {C_{b} |{\mathbf{X}}} \right] = 2trace\left( {{\mathbf{B}}^{2} } \right) = 2\mathop \sum \limits_{{\left( {i,j} \right) \in B}} d_{ij}^{2}$$

and because the pairwise covariances of $V_{g} \left| {{\mathbf{X}},C_{w} } \right|{\mathbf{X}},$ and $C_{b} |{\mathbf{X}}$ are equal to zero (see Eq. A2 in Appendix A),

we get

11 $$var\left[ {V_{A} {|}{\mathbf{X}}} \right] = { }2trace\left( {{\mathbf{D}}^{2} } \right) = 2\mathop \sum \limits_{i = 1}^{L} \mathop \sum \limits_{j = 1}^{L} d_{ij}^{2}$$

While $V_{g}$ is always positive, $C_{w}$ and $C_{b}$ can become negative. In particular, if the QTL effects of the reference allele have an equal chance of being positive or negative, as applies for ${\mathbf{a}} \sim N\left( {0,{\mathbf{I}}} \right),$ there is a higher probability of observing more negative than positive genetic covariances among QTL pairs (see Appendix B) and the distributions of $V_{A} |{\mathbf{X}}$, $C_{w} |{\mathbf{X}}$, and $C_{b} |{\mathbf{X}}$ show a positive skewness (Suppl. Figs. S1 and S2).

To allow comparisons across simulation scenarios differing in the number of QTL and in allele frequencies at QTL, we quantify the contribution of the components ${C}_{w}$ and ${C}_{b}$ to ${V}_{A}$ relative to the contribution of ${V}_{g}$, which can be expressed by the ratios ${b}_{w}=\frac{{C}_{w}}{{V}_{g}}$, ${b}_{b}=\frac{{C}_{b}}{{V}_{g}}$ and $b=\frac{{C}_{w}+{C}_{b}}{{V}_{g}}={b}_{w}+{b}_{b}$.

Since the covariances of $b_{w} |{\mathbf{X}}$ and $b_{b} |{\mathbf{X}}$ are approximately zero (see Eq. A3 in Appendix A) we get

12 $${{var}} \left[ {b{|}{\mathbf{X}}} \right] \approx {{var}} [b_{w} |{\mathbf{X}}] + {{var}} [b_{b} |{\mathbf{X}}]$$

Using properties of $V_{g} |{\mathbf{X}}$, $C_{w} |{\mathbf{X}}$ and $C_{b} |{\mathbf{X}}$, given in Eqs. A4, A5, A6, A7 in Appendix A, we get

13 $$E\left[ {b_{w} |{\mathbf{X}}} \right] \approx 0, E\left[ {b_{b} |{\mathbf{X}}} \right] \approx 0 , {\text{ and }}\;E\left[ {b|{\mathbf{X}}} \right] \approx 0$$

14 $$ {{var}} \left[ {b_{w} |{\mathbf{X}}} \right] \approx \frac{{2{{trace}}\left( {{\mathbf{W}}^{2} } \right)}}{{\left( {{{trace}}\left( {\mathbf{V}} \right)} \right)^{2} }}$$

15 $$ {{var}} \left[ {b_{b} |{\mathbf{X}}} \right] \approx \frac{{2{{trace}}\left( {{\mathbf{B}}^{2} } \right)}}{{\left( {{{trace}}\left( {\mathbf{V}} \right)} \right)^{2} }}$$

16 $$ {{var}} \left[ {b|{\mathbf{X}}} \right] \approx \frac{{2{{trace}}\left( {{\mathbf{W}}^{2} } \right) + 2{{trace}}\left( {{\mathbf{B}}^{2} } \right)}}{{\left( {{{trace}}\left( {\mathbf{V}} \right)} \right)^{2} }}$$

Values of $b_{w}$ and $b$ are restricted to $\ge - 1$. In contrast, $b_{b}$ can be smaller than − 1 if $b_{w} > 1$ (for an example, see Appendix C). If all QTL pairs have a positive genetic covariance, then $b_{w}$, $b_{b}$, and $b$ assume their maximum, which can exceed 1 by far.

Provided the population size used for random mating is sufficiently large, then GPD and $d_{ij}$ values of unlinked QTL $i$ and $j$ are expected to decrease at a rate of $\frac{1}{2}$ per generation (Falconer and Mackay 1996). Thus, from Eq. 15, we obtain that ${{var}} \left[ {b_{b} |{\mathbf{X}}} \right]$ is reduced by a factor of $\frac{1}{4}$. Moreover, the expectation and the shape of the distribution of $b_{b} |{\mathbf{X}}$ will not be altered by $r$ generations of random mating except for the reduction in its variance by a constant factor $\frac{1}{{4^{r} }}$. Due to the restricted recombination between QTL pairs on the same chromosomes, the variance of $b_{w} |{\mathbf{X}}$ is reduced at most by a factor of $\frac{1}{4}$ per generation.

### Genetic material

We used experimental genotypic data from maize (*Zea mays* L.) to simulate the GPD present in two types of ancestral populations. The first ancestral population, called Elite, consisted of a subset of 115 Flint lines from the maize breeding program at the University of Hohenheim (Schrag et al. 2019). The lines were genotyped with the Illumina SNP chip MaizeSNP50 (Ganal et al. 2011) and quality checks as well as imputation were performed as described by Technow et al. (2014), resulting in 38,119 SNPs.

The second ancestral population, called Landrace, was a random sample of 115 DH lines of the 409 DH lines derived from the Landrace Petkuser Ferdinand Rot described by Hölker et al. (2019, 2022). They were genotyped with the 600k Affymetrix© Axiom© Maize Array (Unterseer et al. 2014) with quality checks as described in Mayer et al. (2017, 2020, 2022) resulting in 501,124 SNPs. A genetic map generated from an F_2_ mapping population of the cross EP1 x PH207 (Haberer et al. 2020) was used to assign genetic positions to the 27,542 SNPs which were polymorphic across both ancestral populations, overlapped between both SNP chips, and covered in total 1442 cM of the maize genome.

A total of 2500 SNPs were randomly chosen from the set of 27,542 SNPs polymorphic across both ancestral populations as potential QTL positions with the restriction that on each chromosome their number was proportional to its genetic length.

With the 2500 SNPs used as potential QTL positions, we performed an AMOVA (Excoffier et al. 1992) across all 230 inbred lines derived from both ancestral populations to determine the molecular variance within and among them. Within each ancestral population, we estimated LD as $r^{2}$ between all pairs of potential QTL positions on each chromosome following Hill and Robertson (1968). We estimated the decay of $r^{2}$ with genetic distance based on nonlinear regression according to Hill and Weir (1988) using a threshold of $r^{2} = 0.1$ to quantify the LD decay distance.

For simulating traits, out of the 2500 SNPs we randomly sampled $L$ QTL with effects ${ }{\mathbf{a}}$ of the reference alleles from ${ }{\mathbf{a}} \sim N\left( {0,{\mathbf{I}}} \right).$

### Simulation setup and analysis

#### Simulation of segregating populations

**Fig. 1:**
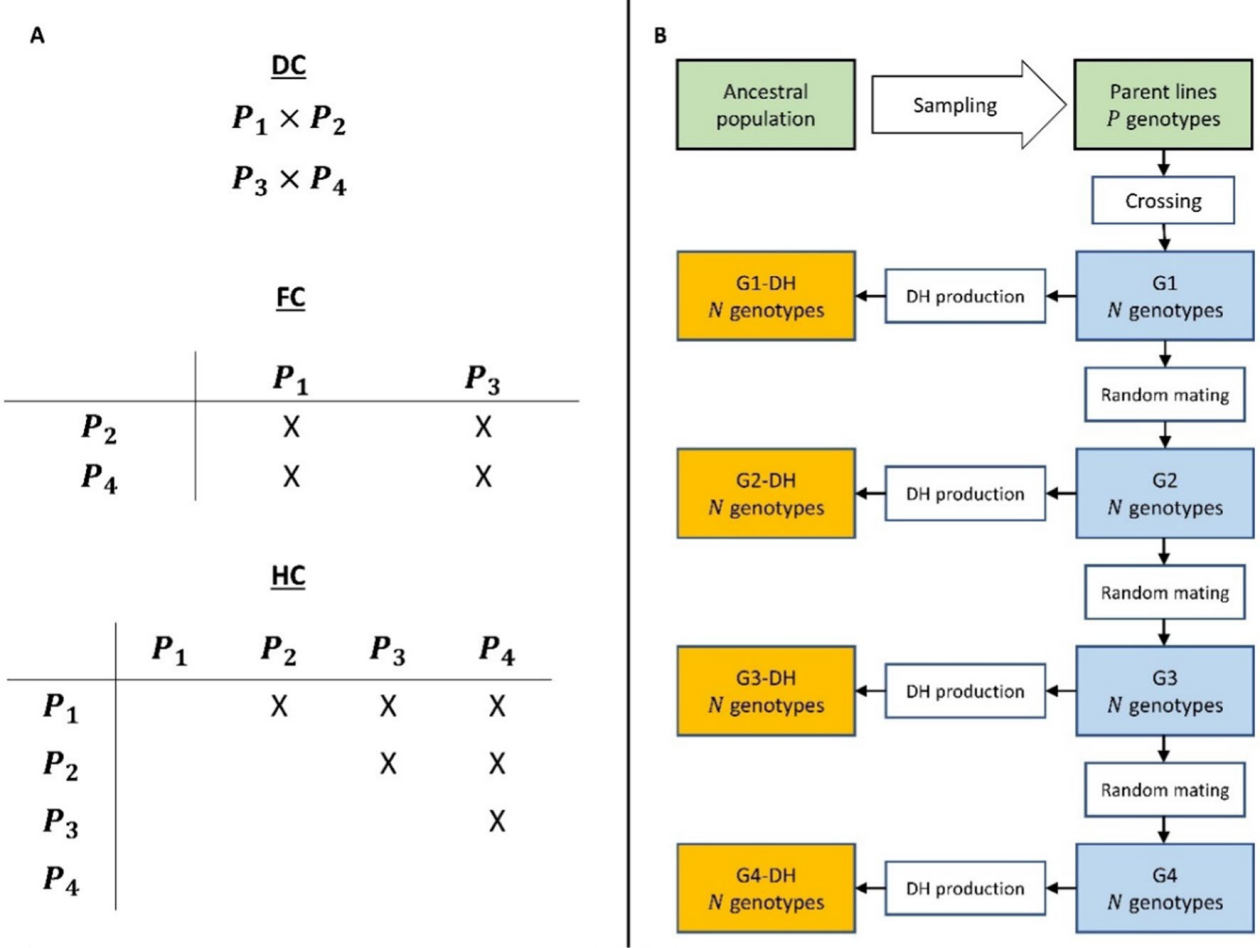
(**A**) Crossing schemes of the three mating designs (disjoint cross (DC), factorial cross (FC), and half-diallel cross (HC)) exemplified with four parental lines (${P}_{1},{P}_{2},{P}_{3},{P}_{4}$). (**B**) Flowchart for one replication of the simulation, starting with the sampling of $P \in \{\mathrm{2,4},\mathrm{8,16}\}$ parental lines and $N \in \{50, 250, 1000\}$ genotypes in the simulated populations. G1, G2, G3, G4 refer to the generation of intermating and G1-DH, G2-DH, G3-DH, G4-DH to the DH populations derived from the respective generation

For a given ancestral population, sets of $P \in \left\{ {2, 4, 8, 16} \right\}$ parental lines were sampled at random. For *P* = 2, the parental lines were crossed to generate a single biparental population. For *P* > 2, the parental lines were intermated according to three different mating designs depicted in Fig. 1A to produce generation G1. For the disjoint cross (DC) design, biparental progenies were generated by ordering the parental lines randomly and crossing the first line with the second, the third with the fourth, and so forth, to produce $\frac{P}{2}$ disjoint crosses. For the factorial cross design (FC), the parental lines were randomly divided into two sets and all possible crosses between the two sets were made leading to $\left( \frac{P}{2} \right)^{2}$ crosses. For the half-diallel cross design (HC), all possible $\frac{{P\left( {P - 1} \right)}}{2}$ crosses were produced. For each mating design, $F_{1}$ progenies were randomly sampled with replacement from the crosses to a total of $N \in \left\{ {50, 250, 1000} \right\}$ genotypes. For *P* = 2, generation G1 is derived from one biparental cross and, hence, comprises $N$ genetically identical $F_{1}$ genotypes. For* P* > 2, generation G1 represents $N$ $F_{1}$ genotypes randomly sampled from the $\frac{P}{2}$, $\left( \frac{P}{2} \right)^{2}$, or $\frac{{P\left( {P - 1} \right)}}{2}$ crosses conditional on the applied mating design. From each genotype in G1, one DH line was generated to produce generation G1-DH. In addition, the $N$ genotypes in G1 were randomly mated (excluding selfing) to obtain $N$ individuals in generation G2. For *P* = 2 this is equivalent to generating the $F_{2}$ generation of a biparental cross. Random mating with $N$ genotypes was continued until generation G4. In parallel, one DH line was produced from each of the $N$ individuals used for random mating in generation G2, G3 and G4 to produce generations G2-DH, G3-DH, and G4-DH (Fig. 1B).

#### Validation of theoretical results

Theoretical derivations in Eqs. 6–16 were validated with simulations as follows. For a given matrix ${\mathbf{X}}$, determined at random from one set of QTL positions in one replication of G1-DH with $P \in \left\{ {2,4} \right\}$, $N = 1000$, and $L = 1000$ QTL, using Elite as the ancestral population and DC as the mating design, we calculated for 10,000 samples of $\user2{a }\sim \user2{ }N\left( {0,{\mathbf{I}}} \right)$ the realized values of $V_{g} |{\mathbf{X}}$, $V_{g} |{\mathbf{X}}$ + $C_{w} |{\mathbf{X}}$, $V_{g} |{\mathbf{X}}$ + $C_{b} |{\mathbf{X}}$**,** $V_{A} |{\mathbf{X}}$ (Suppl. Fig. S1) and $C|{\mathbf{X}}$** = **$C_{w} |{\mathbf{X}}$ + $C_{b} \left| {{\mathbf{X}}, C_{w} } \right|{\mathbf{X}}$, $C_{b} |{\mathbf{X}}$ (Suppl. Fig. S2A) as well as their means, variances, and the skewness and kurtosis of the distribution of the 10,000 realizations and compared them with the corresponding values obtained from theory.

#### Parameter combinations

We defined a “scenario” as the combination of ancestral population (Elite or Landrace), mating design (DC, FC, or HC), and choice of $P \in \left\{ {2, 4, 8, 16} \right\}$, $N \in \left\{ {50, 250, 1000} \right\}$, and $L \in \left\{ {50, 250, 1000} \right\}.$ For each scenario we simulated 500 replications. A “replication” was defined as a simulation run starting from the sampling of the parental lines and either generating a biparental population or applying the chosen mating design for producing in silico the four generations G1-DH to G4-DH. In every replication 50 sets of $L$ QTL positions out of the pool of 2500 potential positions were sampled to obtain 50 realizations of the matrix ${\mathbf{X}}$ comprising the genotypic scores at the QTL, resulting in 25,000 realizations of ${\mathbf{X}}$ per scenario.

Estimates of $E\left[ {V_{A} } \right]$, ${ }var\left[ {V_{g} } \right]$, $var\left[ C \right]$, $var\left[ {C_{w} } \right]$, $var\left[ {C_{b} } \right]$, $var\left[ {V_{A} } \right]$, $var\left[ {b_{w} } \right]$, $var\left[ {b_{b} } \right]$, and $var\left[ b \right]$ were obtained for each scenario by averaging $E\left[ {V_{A} |{\mathbf{X}}} \right] , var\left[ {V_{A} {|}{\mathbf{X}}} \right]$, and the other statistics, calculated for given ${\mathbf{X}}$ according to Eqs. 7–16, over the 25,000 realizations of ${\mathbf{X}}$.

As mentioned above, under infinite population size ($N$ = $\infty$), $var\left[ {b_{b} } \right]$ is expected to be reduced by a factor $\frac{1}{4}$ for every recombination step if the population size is sufficiently large. To describe $var\left[ {b_{b} } \right]$ after $r$ recombination steps with a finite population size $N$ in all generations, denoted as $\widehat{var[{b}_{b}|N]_{r}}$, we used the regression model

17 $$\widehat{var[{b}_{b}|N]_{0}} = \theta + \omega {\text{ and }} \widehat{var[{b}_{b}|N]}_{r+1} = \frac{1}{4} \widehat{var[{b}_{b}|N]}_{r} + \omega $$

where ${\uptheta } = {{var[}}b_{b} {|}N = \infty ]$ under an infinite population size and ${\upomega }$ is the deviation due to sampling from a constant finite population in all generations. Solving the recursive formula, we obtain

18 $$\widehat{var[{b}_{b}|N]_{r}} = \frac{1}{{4^{r} }}\theta + \left( {\frac{1}{{4^{0} }} + \frac{1}{{4^{1} }} + \frac{1}{{4^{2} }} + ... + \frac{1}{{4^{r} }}} \right)\omega = \frac{1}{{4^{r} }}\theta + \left( {\frac{{4 - \frac{1}{{4^{r} }}}}{3}} \right) \omega$$

We used a nonlinear least squares regression implemented in the function *nls*() from the R-package *stats* to estimate ${\upomega }$ in every scenario starting in G1-DH using Eq. 18 (R Core Team 2019).

Recombination of lines was simulated with R package *AlphaSimR v1.1.2* setting the crossover interference parameter to 1 according to Haldane’s mapping function (Faux et al. 2016; Gaynor et al. 2020; Haldane 1919). All other simulations were performed with customized R scripts.

## Results

Out of the 2500 potential QTL positions, 4.9% and 16.3% were monomorphic in ancestral population Elite and Landrace, respectively. The majority (64.9%) of the molecular variance in the AMOVA was within populations (Suppl. Table S1), with Landrace having a higher molecular variance than Elite due to a larger proportion of loci with high minor allele frequency (Suppl. Fig. S3). The LD decay distance was similar for Landrace (21.3 cM) and Elite (22.2 cM).

Estimates of $E\left[{V}_{A}|\mathbf{X}\right]$ and $var\left[{V}_{A}|\mathbf{X}\right]$ obtained from 10,000 realizations of $\mathbf{a}$ conditional on one realization of $\mathbf{X}$ matched very closely the expectations from theory (Suppl. Fig. S1). For *P* = 2, $var\left[{V}_{A}|\mathbf{X}\right]$ was mainly driven by ${C}_{w}|\mathbf{X}$. For* P* = 4, $var\left[{V}_{A}|\mathbf{X}\right]$ was driven to some extent by ${C}_{w}|\mathbf{X}$ but even more by ${C}_{b}|\mathbf{X}$ and this contributed to its pronounced positive skewness and leptokurtic distribution. The distributions of ${b}_{w}|\mathbf{X}$ and ${b}_{b}|\mathbf{X}$ had the same skewness and kurtosis as ${C}_{w}|\mathbf{X}$ and ${C}_{b}|\mathbf{X}$ (Suppl. Fig. S2).

**Fig. 2:**
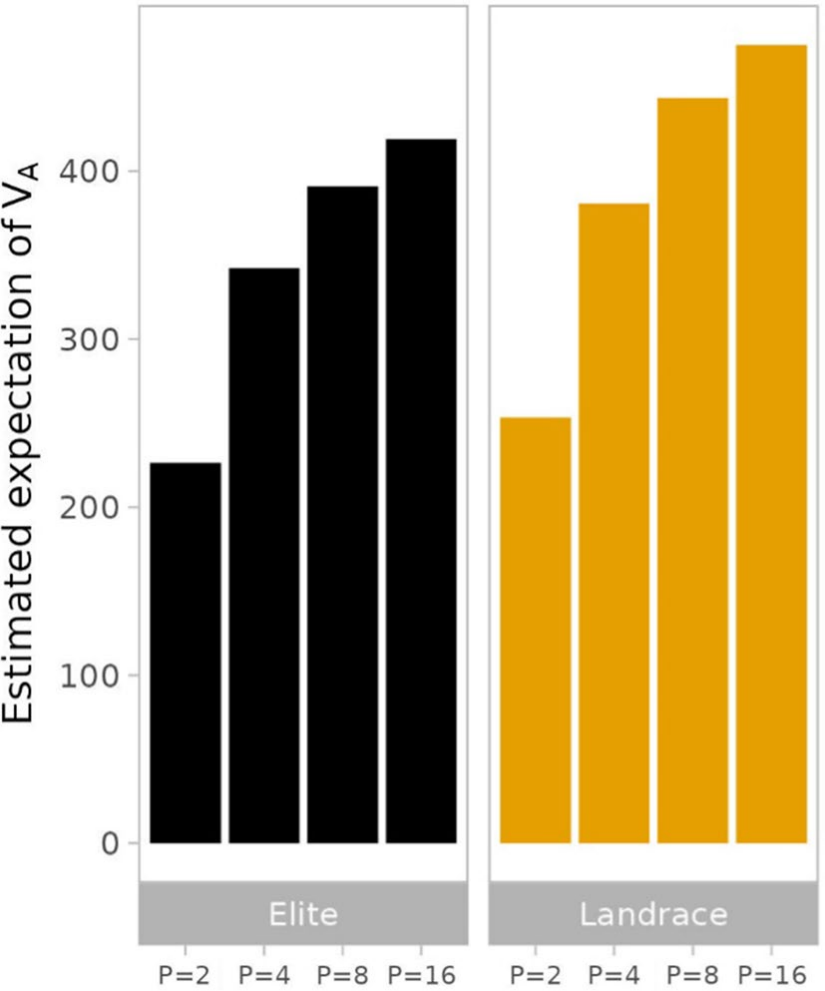
Expectation of ${V}_{A}$ estimated for different numbers of parental lines $P \in \{\mathrm{2,4},\mathrm{8,16}\}$ in generation G1-DH sampled from ancestral population Elite (black) and Landrace (yellow) in scenarios with* N* = 1000 genotypes and *L* = 1000 QTL

As expected from theory, $\widehat{E\left[{V}_{A}\right]}$ (being = $\widehat{E\left[{V}_{g}\right]}$) showed no differences between the mating designs and increased linearly with $L$, because $E[{V}_{g}]$ depends solely on the expected allele frequencies and the sum across QTL. Figure 2 shows a substantial increase (~ 86%) in $\widehat{E\left[{V}_{A}\right]}$ from $P=2$ to $P=16$ for both ancestral populations with slightly higher values for Landrace than Elite. As expected, additional recombination steps and choice of $N$ had no effect on $\widehat{E\left[{V}_{A}\right]}$.

For generation G1-DH and scenario $N=1000$ and $L=1000$, $\widehat{var\left[{V}_{A}\right]}$ was mainly determined (~ 95%) by $\widehat{var\left[C\right]}$ with only minor contributions of $\widehat{var\left[{V}_{g}\right]}$ and slightly higher values for Landrace than Elite (Suppl. Fig. S4). The contribution of $\widehat{var\left[{C}_{w}\right]}$ to $\widehat{var\left[C\right]}$ decreased moderately with increasing $P$ for both ancestral populations irrespective of the mating design. By comparison, the contribution of $\widehat{var\left[{C}_{b}\right] }\mathrm{to }\;\widehat{var\left[C\right]}$ was much higher, especially in ancestral population Landrace and mating designs DC and FC, and decreased strongly for larger $P$ values in all scenarios.

**Fig. 3:**
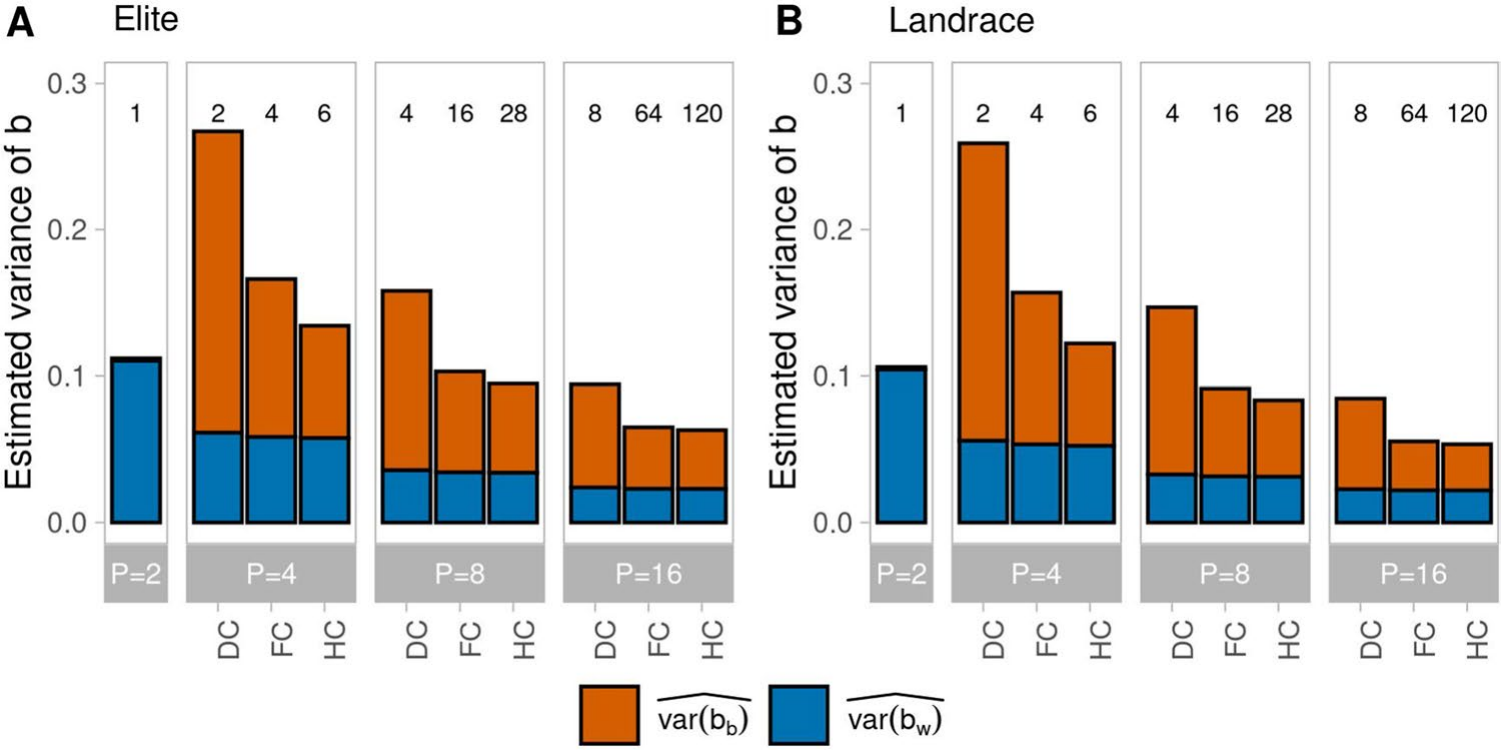
Estimated variance of *b* decomposed into the parts attributable to QTL pairs on different chromosomes ($\widehat{var\left[{b}_{b}\right]}$, red) and on the same chromosome ($\widehat{var\left[{b}_{w}\right] }$, blue) in generation G1-DH for different numbers of parental lines $P \in \{\mathrm{2,4},\mathrm{8,16}\}$ sampled from ancestral population Elite (**A**) and Landrace (**B**) and using three mating designs (disjoint cross (DC), factorial cross (FC), and half-diallel cross (HC)) in scenarios with *N* = 1000 genotypes and *L* = 1000 QTL. The number of crosses generated in the respective mating design is shown above the bars

This pattern carried over to $\widehat{var\left[b\right]}$ and its components, where $\widehat{var\left[{b}_{b}\right]}$ contributed substantially more than $\widehat{var\left[{b}_{w}\right]}$ for $P>2$ (Fig. 3), but estimates were slightly smaller for ancestral population Landrace due to the larger values of ${(\widehat{E\left[{V}_{g}\right])}}^{2}$ in the denominator of the formulas in Eq. 14 and 15. Increasing $P$ from 4 to 16 lead to a substantial reduction of $\widehat{var\left[{b}_{w}\right]}$ and even more so of $\widehat{var\left[{b}_{b}\right]}$, so that $\widehat{var\left[b\right]}$ was reduced by 38 to 67% for all scenarios. While for given $P>2, \widehat{var\left[{b}_{w}\right]}$ changed only slightly from DC to FC and HC, $\widehat{var\left[{b}_{b}\right]}$ was by far largest for mating design DC, followed by FC and HC, with smaller differences for larger values of $P$. For $P=2,$ $\widehat{var\left[{b}_{b}\right]}$ was small for $N$ = 1000 and $\widehat{var\left[{b}_{w}\right]}$ was higher compared to all other scenarios with $P>2$.

**Fig. 4:**
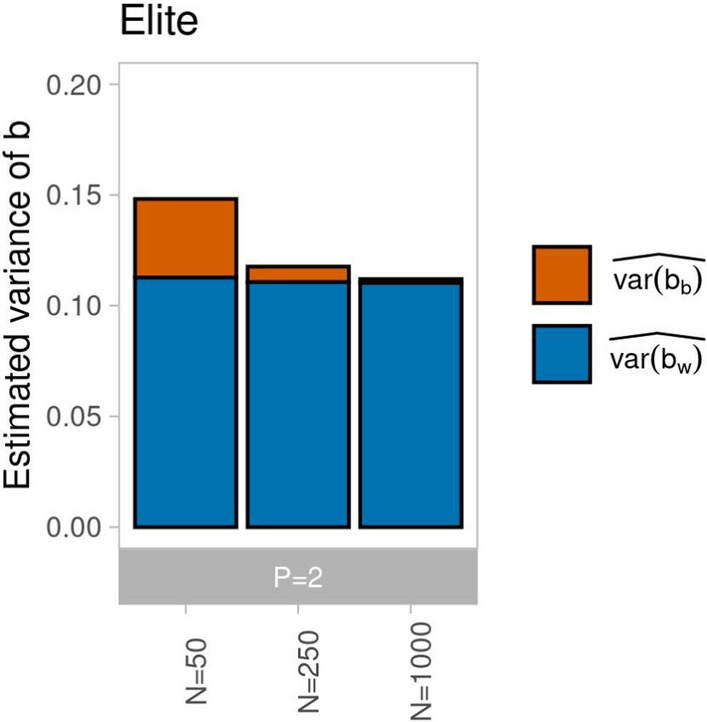
Estimated variance of *b* decomposed into the parts attributable to QTL pairs on different chromosomes ($\widehat{var\left[{b}_{b}\right]}$, red) and on the same chromosome ($\widehat{var\left[{b}_{w}\right] }$, blue) in generation G1-DH using *P* = 2 parental lines sampled from ancestral population Elite and varying $N \in \{50, \mathrm{250,1000}\}$ of genotypes and *L* = 1000 QTL

For DH lines derived from a single cross, with finite sample size $N$ the GPD between loci on different chromosomes can differ from zero, the value expected for $N=\infty$. We analyzed for $P=2$ the effect of $N$ on the magnitude and composition of $\widehat{var\left[b\right]}$ in generation G1-DH for ancestral population Elite (Fig. 4). While $\widehat{var\left[{b}_{w}\right]}$ remained constant, the contribution of $\widehat{var\left[{b}_{b}\right]}$ amounted to 24%, 6%, and 2% of $\widehat{var\left[b\right]}$ for $N=50$, $250$, and 1000, respectively.

Reducing the number of QTL from $L=1000$ to $250$ and 50 decreased $\widehat{var\left[b\right]}$ by 2–6 and 7–24%, respectively, for all scenarios and did not alter the relative contributions of $\widehat{var\left[{b}_{b}\right]}$ and $\widehat{var\left[{b}_{w}\right]}$ (Suppl. Fig. S5), which depended on $P$ and the mating design (Fig. 3).

**Fig. 5:**
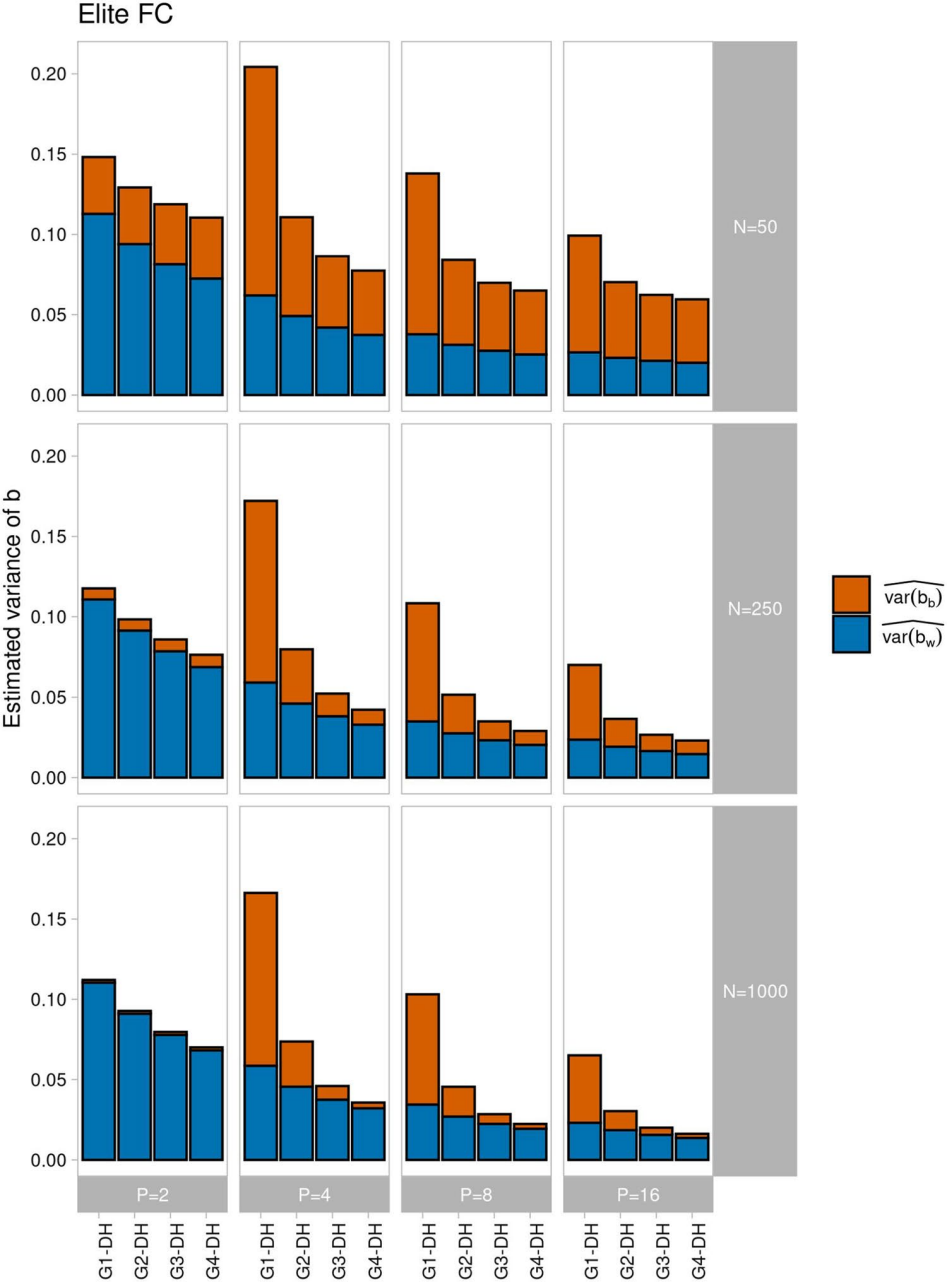
Estimated variance of **b** decomposed into the parts attributable to QTL pairs on different chromosomes ($\widehat{var\left[{b}_{b}\right]}$, red) and on the same chromosome ($\widehat{var\left[{b}_{w}\right] }$, blue) in generations G1-DH to G4-DH for different numbers of parental lines $P \in \{\mathrm{2,4},\mathrm{8,16}\}$ sampled from ancestral population Elite using the mating design factorial cross (FC) for producing generation G1 and $N \in \{50, 250, 1000\}$ genotypes for producing generations G1 to G4 and G1-DH to G4-DH and *L* = 1000 QTL

According to theory (Eq. 18), intermating with $N= \infty$ is expected to reduce $\widehat{var\left[{b}_{b}\right]}$ by $\frac{1}{4}$ per generation. Our simulations for ancestral population Elite and mating design FC fit this expectation well for $N=1000$, but the decay was much slower for $N=50$ (Suppl. Fig. S6). The parameter $\omega$ describing the effect of finite population size in the nonlinear regression model (Eq. 18) was negligible for $N \ge 250$ ($\omega \le 0.006$) but became significant for $N=50$ ($\omega =0.03$). As a consequence of the GPD generated anew in each generation by using a finite $N$, which counteracts the reduction in GPD due to intermating, $\widehat{var\left[{b}_{b}\right]}$ did not fully decay so that in generation G4-DH a kind of steady state was approached, the level of which depended strongly on $N$ but was independent of $P$ (Fig. 5). For $P=2$, $\widehat{var\left[{b}_{b}\right]}$ attributable to finite $N$ was nearly constant from generations G1-DH to G4-DH and sizeable for $N=50$. The reduction in $\widehat{var\left[{b}_{w}\right]}$ with progressing intermating followed a linear relationship with a weak convex curvature and was largely independent of $N$, yet the level was about four times higher for $P=2$ than for $P=16$.

**Fig. 6:**
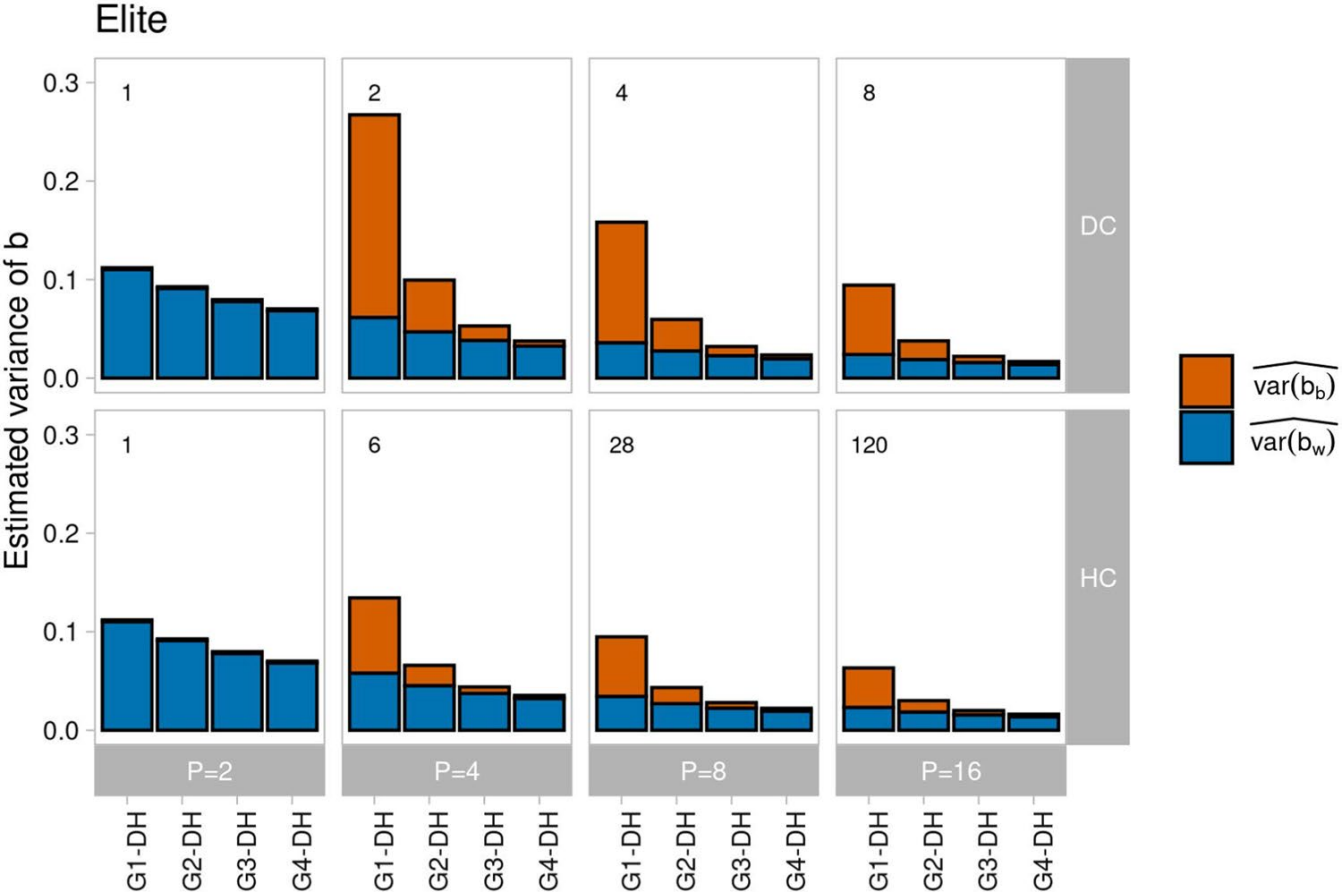
Estimated variance of *b* decomposed into the parts attributable to QTL pairs on different chromosomes ($\widehat{var\left[{b}_{b}\right]}$, red) and on the same chromosome ($\widehat{var\left[{b}_{w}\right] }$, blue) in generations G1-DH to G4-DH for different numbers of parental lines $P \in \{\mathrm{2,4},\mathrm{8,16}\}$ sampled from ancestral population Elite and using the mating designs disjoint cross (DC) and half-diallel cross (HC) in scenarios where generation G1 and generations G1 to G4 and G1-DH to G4-DH are produced with *N* = 1000 genotypes and *L* = 1000 QTL. The number of crosses generated in the respective mating design is shown above the bars

The mating design neither affected the level nor the rate of reduction of $\widehat{var\left[{b}_{w}\right]}$ in generations G1-DH to G4-DH (Figs. 5 and 6). By contrast, the initial level of $\widehat{var\left[{b}_{b}\right]}$ was almost twice as large for mating design DC as for HC and intermediate for FC. Altogether, the reduction in $\widehat{var\left[b\right]}$ was most effective in the first intermating generation but the efficacy depended on the mating design, the number of parents, and the sample size employed for generating and intermating the population from which the DH lines were derived. For the special case $P=2$, intermating reduced $\widehat{var\left[b\right]}$ only at a low rate, especially if $N$ was small.

## Discussion

The additive genetic variance ${V}_{A}$ inherent to a breeding population is a major determinant of short- and long-term genetic gain. Consequently, it is crucial for breeders to have reliable estimates of ${V}_{A}$ among selection candidates and strategies of intervention if the variance is depleted by selection. Allier et al. (2019b) used phenotypic and molecular data for a temporal analysis of ${V}_{A}$ in a North European grain maize breeding program and found that on a whole-genome basis, negative covariances between QTL masked about one fourth of the genic variance making it inaccessible to selection. In a simulation study, Lara et al. (2022) obtained similar results from the analysis of a wheat breeding program. They concluded that negative covariances of QTL pairs on different chromosomes were a major force that affected the change in ${V}_{A}$ across selection steps within the same breeding cycle and across cycles. Here, we investigated how mating designs and the number of parents affect the variation in ${V}_{A}$ among breeding populations. In the following we discuss how this relates to the success of phenotypic and genome based-selection.

### Mating designs have a strong influence on the observed additive genetic variance

The observed $V_{A}$ in a breeding program is subject to sampling. Variation in $V_{A}$ arises as different realizations of ${\mathbf{X}}$ lead to variation in QTL allele content. We developed a theoretical framework to assess the dispersion of $V_{A}$ and its components around their respective expected values. In addition, we calculated the variance of $V_{A}$ and of the parameter $b$ (covariance component $C$ in $V_{A}$ standardized by $V_{g}$) for different mating designs and number of parents in simulated data.

Our results show that it is mainly the covariance component $C$ that contributes to differences in the variance of $V_{A}$ among mating designs (Suppl. Fig. S4), as allele frequencies of the progenies (e.g. in G1-DH), which determine the genic variance $V_{g}$, are not affected by the design. While the mating design affected mainly the between chromosome covariance component, the number of parents had an effect on both, variation in covariances of QTL pairs on the same and on different chromosomes. When the number of crosses was constant (e.g. DC, $P$ = 8 and FC, $P$ = 4), differences between mating designs were alleviated when the covariance component $C$ was expressed relative to the genic variance $V_{g}$ (Fig. 3). In general, variation in *b* was highest for populations comprising large biparental families derived from few disjoint crosses, thus the DC design carries a high risk of generating progenies with deflated or inflated $V_{A}$. The consequences would be reduced selection gain due to masking of the genic variance by an excess of negative QTL covariances or an overly optimistic assessment of future selection gains due to an inflated $V_{A}$ arising from an excess of positive QTL covariances.

The three designs analyzed in this study are stylized examples of crossing schemes, but in practice the number of crosses and progenies per cross vary not only between breeding programs but also within the same program across selection cycles (Auinger et al. 2021). Therefore, the inferences from Fig. 3 are recommended as general guidelines. If breeders are aware that observed values of $V_{A}$ vary between samples, especially when large families are generated from few disconnected crosses, they can take interventions if necessary. Already one round of recombination can substantially mitigate under- or overestimation of $V_{g}$ by breaking negative and positive covariances between QTL pairs and reducing the between chromosome covariance component of $V_{A}$ to a large extent (Figs. 5 and 6). If the additional time needed for recombination is compensated by higher selection gain due to increased $V_{A}$ will be crop and program specific and warrants further research. Nevertheless, with genomic data at hand, it might be advisable for breeders to obtain estimates of the genomic variance from breeding populations with statistical methods differing in their treatment of QTL covariances and informing about the difference between $V_{g}$ and $V_{A}$ as suggested by Lehermeier et al. (2017a) and Allier et al. (2019b).

The results from this study also allow inferences about the suitability of the different mating designs for genome-based prediction using ridge regression BLUP as statistical method. For ridge regression BLUP, it is known that estimates of marker effects are strongly affected by the so-called grouping effect (Zou and Hastie 2005). An upper bound exists for pairwise differences between estimated marker effects, which is a function of their correlation coefficient and the extent of regularization. Thus, even if QTL lie on different chromosomes and their true allelic effects differ, their effect estimates are equalized by the model if their genotypic scores are highly correlated (for an example see Lehermeier et al. 2017a). A mating design like DC carries a high risk of producing data sets in which $V_{A}$ is dominated by the between chromosome covariance component. When training a genome-based prediction model on a subset of progenies with phenotypes from such a data set and predicting the remaining progenies without phenotypes from the same population sample (i.e. within cycle prediction), the accuracy of prediction should not be compromised by the population structure as long as QTL covariances are consistent across training and prediction set. However, if $V_{A}$ is decreased due to negative QTL covariances, the prediction accuracy in the respective sample might be low as the accuracy is a function of trait heritability (Daetwyler et al. 2008). In addition, when using the model to predict genetic values of the next breeding cycle (i.e. across cycle prediction), recombination will have changed QTL covariances dramatically and the prediction accuracy is likely to break down. One way to mitigate this effect of the QTL covariance structures in the training population is to train the model on data from several breeding cycles and years as suggested by Auinger et al. (2016, 2021), but genome-based prediction accuracy might also be compromised if a population sample exhibiting strong QTL covariances is used as prediction set. Auinger et al. (2021) reported in their study that expected and observed prediction accuracy differed strongly for one of two prediction sets. They concluded that this might have been the result of low effective sample size and high linkage disequilibrium, both pointing to a high probability of strong covariances between QTL. How to mitigate the effects of variation in the covariance component among prediction sets in genome-based selection has not been solved, but it is certainly an interesting subject of future research which mating designs will maximize the success of genome based selection, especially if rapid cycling selection without model retraining is employed.

### Variation of ${\varvec{V}}_{{\varvec{A}}}$ and the usefulness criterion

If the focus in a breeding program lies on short-term selection gain, breeders often generate large biparental families derived from crosses of a few “best” parents. In such a scenario it might be rewarding to apply the usefulness criterion (Schnell and Utz 1975), i.e. to ensure that the selected parents produce progenies with high mean performance and high $V_{A}$. In the context of genome based breeding, molecular data can be used to predict not only the mean genetic value of a cross, but also $V_{A}$ among its progenies. Genetic values of progenies of a cross are either simulated based on parental genotypes and information on map distances (e.g. Mohammadi et al. (2015)) or by using analytical solutions for specific types of crosses as presented by Lehermeier et al. (2017b) for biparental families and extended by Allier et al. (2019a) for more complex crosses. The resulting genetic values will allow prediction of the mean genetic value of the respective cross and its variance. Both, the in silico and the analytical approach assume absence of GPD between QTL on different chromosomes. This assumption is justified for very large biparental populations ($N$ > 250), but our results show that QTL covariances between chromosomes pertain up to $N$ = 250 and contribute substantially to the variance of $b$ for smaller biparental populations (24% for $N$ = 50 and $L$ = 1000 with Elite as the ancestral population, Fig. 4). Thus, in addition to imperfect information on the genetic distances between markers in a specific cross, covariances between QTL on different chromosomes are likely to reduce the effectiveness of the usefulness criterion in comparison to selection on the predicted progeny mean. In lines derived from disjoint four-way crosses as assumed in Allier et al. (2019a), variation in covariances between QTL on different chromosomes are expected to be even more pronounced than in biparental crosses (Fig. 5). In a multi-trait context this effect is also exacerbated. In a given cross, the QTL for trait 1 might generate a different covariance pattern as the QTL for trait 2 affecting the respective estimates of $V_{A}$ and corresponding trait covariances. Additional recombination can mitigate the effect, but only if the number of progenies derived from each cross is sufficiently large (Fig. 5). These results are corroborated by findings from outcrossing species. While Iwata et al. (2013) found very good agreement of predicted and observed $V_{A}$ in a large biparental cross of Japanese pear (*N* = 1000), Wolfe et al. (2021) found only low prediction accuracies for $V_{A}$ in Cassava, most likely due to small family sizes.

### Sign of the covariance component

The variance of $V_{A}$ for a given trait and population sample depends on the vector ${\mathbf{a}}$ reflecting the size, sign, and phase of QTL effects and on the realization of the matrix ${\mathbf{X}}$ reflecting the allele content at QTL and the magnitude of GPD between QTL pairs. We validated our theoretical solutions with respect to $var\left[ {V_{A} {|}{\mathbf{X}}} \right]$ and its components by simulating 10,000 samples of the vector of QTL effects ${\mathbf{a}}$ conditional on one realization of ${\mathbf{X}}$. Theoretical and simulated results for the expectation and variance of $var\left[ {V_{A} {|}{\mathbf{X}}} \right]$ were highly congruent. Moreover, simulation results show nicely that the distribution of QTL covariances is not symmetric (Suppl. Figs. S1, S2), because the covariance component $C$ has a lower bound relative to the genic variance $V_{g}$, so that *b* ≥ –1, but *b* can exceed 1 substantially if many QTL pairs have a positive covariance (see Appendix C). For* P* = 4 and mating design DC this was especially obvious for QTL pairs on different chromosomes with some samples of QTL effects resulting in large positive values of $C_{b} .$ Even if the QTL effects are sampled from a symmetric distribution about the origin, then for a given realization of ${\mathbf{X}}$ the difference between positive and negative QTL pair covariances still has a probability > 0.5 to be negative ($P\left[ {Y < 0; L,0.5} \right] > 0.5$, for details see Appendix B) and the distribution of $Y$ will display positive skewness (***γ***_1_ = 4.54, Suppl. Fig. S7A), which carries over to the distributions of $C|{\mathbf{X}}$, $C_{w} |{\mathbf{X}}$, and $C_{b} |{\mathbf{X}}$ (Suppl. Fig. S2A) pointing to a high risk of severely inflated estimates of $V_{A}$ for some QTL samples, i.e. traits.

In both ancestral populations, Elite and Landrace, the GPD values $d_{ij}$ of matrix ${\mathbf{D}}$ were symmetrically distributed around zero (Suppl. Fig. S8). This was expected as allele coding was based on the B73 reference sequence, i.e. more or less at random. As both ancestral populations had similar allele frequencies and linkage disequilibrium, the distributions of GPD values resembled each other for Elite and Landrace and showed that GPD is pervasive in managed populations and needs to be accounted for. However, as pointed out by Lara et al. (2022) nonzero GPD values in matrix ${\mathbf{D}}$ do not necessarily lead to a change in $V_{A}$ as they are trait agnostic and positive and negative QTL covariances can cancel each other when summed across the genome. It is always the combination of the GPD in the population sample and the trait specific allele substitution effects that need to be considered. Our study showed, that even if QTL effects are sampled from a normal distribution, large differences can be observed in the variation of QTL covariances among mating designs. If for some traits QTL are clustered in certain genomic regions and QTL alleles exhibit strong repulsion or coupling linkage, differences between mating designs are likely to become even more pronounced (Appendix C). The same is true if assortative or disassortative crosses are made in contrast to sampling the parents at random from the ancestral populations as done in this study. To investigate these effects warrants further research but would be beyond the scope of this study. We hypothesize that our conclusions with respect to the effect of the mating design and the number of parents on the variation of $V_{A}$ hold across a broad range of scenarios as variation of $V_{A}$ arises mainly from differences in population structure among scenarios.

### Electronic supplementary material

Below is the link to the electronic supplementary material.

Supplementary file1 (PDF 1218 kb)

## Data Availability

The datasets and the scripts for the simulation can be accessed in the GitHub repository https://github.com/TUMplantbreeding/MatingDesignVA.

## Appendices

### Appendix A

For every random vector $\mathbf{x}\sim \left({\varvec{\mu}},{\varvec{\Sigma}}\right)$ and symmetric matrix $\mathbf{A}$, the following result holds true (Mathai and Provost 1992):

A1 $$E\left[ {{\mathbf{x}}^{T} {\mathbf{Ax}}} \right] = trace\left( {{\mathbf{A\Sigma }}} \right) + {\varvec{\mu}}^{T} {\mathbf{A}} {{\varvec{\mu}}}$$

Inserting ${\varvec{\mu}}=0$ and ${\varvec{\Sigma}}=\mathbf{I}$ yields Eq. 7.

If $\mathbf{x}$ has a Gaussian multivariate normal distribution, these authors showed that for two symmetric matrices ${\mathbf{A}}_{1}$ and ${\mathbf{A}}_{2},$ we have

A2 $${\varvec{cov}}\left[ {{\mathbf{x}}^{T} {\mathbf{A}}_{1} {\mathbf{x}},{ }{\mathbf{x}}^{T} {\mathbf{A}}_{2} {\mathbf{x}}} \right] = 2trace\left( {{\mathbf{A}}_{1} {\mathbf{\Sigma A}}_{2} {{\varvec{\Sigma}}}} \right) + 4{\varvec{\mu}}^{T} {\mathbf{A}}_{1} {\mathbf{\Sigma A}}_{2} {{\varvec{\mu}}}$$

from which we obtain Eqs. 8, 9, 10 and also that the covariances of ${V}_{g}|\mathbf{X},{C}_{w}|\mathbf{X},$ and ${C}_{b}|\mathbf{X}$ are zero.

If the random vector $\mathbf{x}$ were sampled from distributions other than the multivariate normal such as the Gamma distribution, one can still derive approximations for the moments of quadratic forms (Mohsenipour and Provost 2013), but more detailed derivations for this case are beyond the scope of this study.

For the derivation that

A3 $$ {{cov}} \left( {b_{w} \left| {{\mathbf{X}},b_{b} } \right|{\mathbf{X}}} \right) \approx 0$$

we use (i) $E\left[{V}_{g}|\mathbf{X}\right]>0$, which implies $E[{\left({V}_{g}|\mathbf{X}\right)}^{2}]>0$, and $E\left[{C}_{w}|\mathbf{X}\right]=0$, $E\left[{C}_{b}|\mathbf{X}\right]=0$ (see Eq. 6), (ii) pairwise covariances of ${V}_{g}|\mathbf{X},{C}_{w}|\mathbf{X},{C}_{b}|\mathbf{X}$ are zero, and (iii) ${cov(V}_{g}\left|\mathbf{X},{C}_{w}\right|\mathbf{X}\times {C}_{b}\left|\mathbf{X}\right)\approx 0$ (supported by simulations), we get

$$\begin{aligned} &  {\text{cov}}\left( {b_{w} \left| {{\mathbf{X}},b_{b} } \right|{\mathbf{X}}} \right) = E\left[ {\frac{{C_{w} |{\mathbf{X}}}}{{V_{g} |{\mathbf{X}}}} \times \frac{{C_{b} |{\mathbf{X}}}}{{V_{g} |{\mathbf{X}}}}} \right] - E\left[ {\frac{{C_{w} |{\mathbf{X}}}}{{V_{g} |{\mathbf{X}}}}} \right]E\left[ {\frac{{C_{b} |{\mathbf{X}}}}{{V_{g} |{\mathbf{X}}}}} \right] \\ & \approx E\left[ {\frac{{C_{w} \left| {{\mathbf{X}} \times C_{b} } \right|{\mathbf{X}}}}{{\left( {V_{g} |{\mathbf{X}}} \right)^{2} }}} \right] - E\left[ {C_{w} |{\mathbf{X}}} \right]E\left[ {\frac{1}{{V_{g} |{\mathbf{X}}}}} \right]E\left[ {C_{b} |{\mathbf{X}}} \right]E\left[ {\frac{1}{{V_{g} |{\mathbf{X}}}}} \right] \\ & \approx E\left[ {C_{w} |{\mathbf{X}}} \right]E\left[ {C_{b} |{\mathbf{X}}} \right]E\left[ {\frac{1}{{\left( {V_{g} |{\mathbf{X}}} \right)^{2} }}} \right] - E\left[ {C_{w} |{\mathbf{X}}} \right]E\left[ {C_{b} |{\mathbf{X}}} \right]\left\{ {E\left[ {\frac{1}{{V_{g} |{\mathbf{X}}}}} \right]} \right\}^{2} \\ & = 0 \times 0 \times E[1/(V_{g} |{\mathbf{X}})^{2} ] - 0 \times 0 \times \left\{E[1/(V_{g} |{\mathbf{X}})\right\}^{2} = 0.\;{\text{q}}.{\text{e}}.{\text{d}}. \\ \end{aligned}$$

We can approximate the expectation and variance of $b|\mathbf{X}$, ${b}_{w}|\mathbf{X}$ and ${b}_{b}|\mathbf{X}$ based on the expectation and variance of ${V}_{g}|\mathbf{X},{C}_{w}|\mathbf{X},$ and ${C}_{b}|\mathbf{X}$ using formulas given by Mood et al. (1974) and obtain

A4 $$E\left[ {b_{w} |{\mathbf{X}}} \right] \approx E\left[ {C_{w} |{\mathbf{X}}} \right]E\left[ {\frac{1}{{V_{g} }}|{\mathbf{X}}} \right] = 0,{ }E\left[ {b_{b} |{\mathbf{X}}} \right] \approx E\left[ {C_{b} |{\mathbf{X}}} \right]E\left[ {\frac{1}{{V_{g} }}|{\mathbf{X}}} \right] = 0,{ }E\left[ {b|{\mathbf{X}}} \right] \approx 0$$

A5 $$ {\text{var}} \left[ {b_{w} |{\mathbf{X}}} \right] = var\left[ {\frac{{C_{w} |{\mathbf{X}}}}{{V_{g} |{\mathbf{X}}}}} \right] \approx \frac{{{{var}} \left[ {C_{w} |{\mathbf{X}}} \right]}}{{\left( {E\left[ {V_{g} |{\mathbf{X}}} \right]} \right)^{2} }} = \frac{{2trace\left( {{\mathbf{W}}^{2} } \right)}}{{\left( {trace\left( {\mathbf{V}} \right)} \right)^{2} }}$$

A6 $$ {\text{var}} \left[ {b_{b} |{\mathbf{X}}} \right] =  {{var}}\left[ {\frac{{C_{b} |{\mathbf{X}}}}{{V_{g} |{\mathbf{X}}}}} \right] \approx \frac{{{{var}} \left[ {C_{b} |{\mathbf{X}}} \right]}}{{\left( {E\left[ {V_{g} |{\mathbf{X}}}\right]} \right)^{2}}}{} = \frac{{2  {{trace}}\left( {{\mathbf{B}}^{2} } \right)}}{{\left({{{trace}}\left( {\mathbf{V}} \right)} \right)^{2} }}$$

and

A7 $$ {\text{var}} \left[ {b|{\mathbf{X}}} \right] \approx {{var}} \left[ {b_{w} |{\mathbf{X}}} \right] + {{var}} \left[ {b_{b} |{\mathbf{X}}} \right] \approx \frac{{2trace\left( {{\mathbf{W}}^{2} } \right) + 2trace\left( {{\mathbf{B}}^{2} } \right)}}{{\left( {trace\left( {\mathbf{V}} \right)} \right)^{2} }}$$

## Appendix B

Let $P\left[{a}_{i}\ge 0\right]=\upbeta$ denote the probability that the allelic effect ${a}_{i}$ of the reference allele at QTL $i$ is positive and $K\in \left\{\mathrm{0,1},\dots ,L\right\}$ the number of QTL with positive sign of the allele effect ${a}_{i}$ in vector $\mathbf{a}$. Then, the number of locus pairs, with allele effects of different signs, is given by $K\left(L-K\right) ,$ and the number of locus pairs, with allele effects of the same sign, is given by $$\left(\begin{array}{c}L\\ 2\end{array}\right)- K\left(L-K\right)$$. Thus, $$Y=\left(\begin{array}{c}L\\ 2\end{array}\right)-2K\left(L-K\right)$$ with $$Y\in \left\{Round\left[-\frac{L}{2}\right],.., \left(\begin{array}{c}L\\ 2\end{array}\right)\right\} (\mathrm{where }\;Round\left[-\frac{L}{2}\right]$$ is the integer nearest to $-\frac{\mathrm{L}}{2}$ rounded toward zero) is the difference in the number of QTL pairs, where the product of allele effects is positive minus the number of QTL pairs, where it is negative. For different realizations of the vector $\mathbf{a}$, $K$ and $Y$ can be regarded as random variables with the probability distributions

$$P\left[K=k|L,\upbeta \right]=\left(\begin{array}{c}L\\ k\end{array}\right){\upbeta }^{k}{\left(1-\upbeta \right)}^{L-k}$$ and

$$P\left[ {\left. {Y = y} \right|K = k;L,\beta } \right] = \left\{ {\begin{array}{*{20}c} 1 & {{\text{if }}y = \left( {\begin{array}{*{20}c} L \\ 2 \\ \end{array} } \right) - 2k\left( {L - k} \right)} \\ 0 & {{\text{elsewhere}}} \\ \end{array} } \right\}$$

so that $$P\left[Y=y;L,\upbeta \right]={\sum }_{k=0}^{L}P\left[\left.Y=y\right|K=k;L,\upbeta \right]P\left[K=k;L,\upbeta \right]$$

$$= \left\{ {\begin{array}{*{20}l} {\sum _{{k \in K\left( y \right)}} \left( {\begin{array}{*{20}c} L \\ k \\ \end{array} } \right)\beta ^{k} \left( {1 - \beta } \right)^{{L - k}} \;{\text{where}}\;K\left( y \right) = \left\{ {\left. k \right|k = \frac{L}{2} \pm \frac{{\sqrt {L + 2y} }}{2} \wedge k \in \left\{ {0,...,L} \right\}} \right\}} \hfill \\ {{\text{or}}\;0\;{\text{if}}\;K\left( y \right) = \emptyset } \hfill \\ \end{array} } \right\}$$

If $\upbeta =0.5,$ as holds true for $\mathbf{a}\sim N\left(0,\mathbf{I}\right)$, we have $P\left[Y<0;\,L=\mathrm{1000,0.5}\right]=0.67,$ which exceeds 0.5 by far (Suppl. Fig. S7B). If for a given population (realization of the matrix $\mathbf{X}$) the distribution of the GPD values ${d}_{ij}$ is symmetric with respect to zero as applies approximately for both ancestral populations (Suppl. Fig. S8), then the properties of the probability distribution for $Y$ carry over to the distributions for ${C}_{w}|\mathbf{X}$ and ${C}_{b}|\mathbf{X}$. Thus, the probability for the sum of QTL covariances being negative is greater than 0.5 and the distributions of ${C}_{w}|\mathbf{X}$, ${C}_{b}|\mathbf{X}$, and especially $C|\mathbf{X}$ exhibit positive skewness (Suppl. Fig. S2A).

## Appendix C

Since ${V}_{A}={V}_{g}+{C}_{w}+{C}_{b}\ge 0$, we get $\frac{{C}_{w}}{{V}_{g}}+\frac{{C}_{b}}{{V}_{g}}\ge \frac{-{V}_{g}}{{V}_{g}}$, from which we obtain $b\ge -1$.

Let ${V}_{A}\left(c\right)$, ${V}_{g}\left(c\right)$, ${C}_{w}\left(c\right)$ denote the additive genetic variance, genic variance and the “within covariance” term for chromosome $c$. Then, from ${V}_{A}\left(c\right)={V}_{g}\left(c\right)+{C}_{w}\left(c\right)\ge 0$, we get ${C}_{w}\left(c\right)\ge -{V}_{g}\left(c\right)$.

Thus, we have ${C}_{w}={\sum }_{c}{C}_{w}\left(c\right)\ge {\sum }_{c}-{V}_{g}\left(c\right)=-{V}_{g}$ so that ${b}_{w}\ge -1$.

We show by the following example that ${b}_{b}$ can be smaller than −1 if ${b}_{w}$ is larger than 1.

Consider a population of DH lines with two chromosomes each having ${L}_{c}$ loci, where only two haplotypes [AAAAA… + bbbbb…] and [aaaaa… + BBBBB…] occur with equal frequency and the additive effects ${a}_{i}=a$ for all QTL. Then the ${d}_{ij}$ values for locus pairs $\left(i,j\right)$ on the same chromosome have a value of 1 (because they are in coupling phase) and QTL pairs $\left(i,j\right)$ on different chromosomes have a value of −1 (because they are in repulsion phase).

Thus, we have ${V}_{g}=2{L}_{c}{a}^{2}$, ${C}_{w}=2{L}_{c}\left({L}_{c}-1\right){a}^{2}$, ${C}_{b}=-2{{L}_{c}}^{2}{a}^{2}$.

As a check, we get ${V}_{A}=2{L}_{c}{a}^{2}+2{L}_{c}\left({L}_{c}-1\right){a}^{2}-2{{L}_{c}}^{2}{a}^{2}=0$, in agreement with the fact that the genotypic values of all DH lines are equal to zero. Consequently, we have

$${b}_{w}={C}_{w}/{V}_{g}=2{L}_{c}\left({L}_{c}-1\right){a}^{2}/2{L}_{c}{a}^{2}=\left({L}_{c}-1\right)$$

$${b}_{b}={C}_{b}/{V}_{g}=-2{{L}_{c}}^{2}{a}^{2}/2{L}_{c}{a}^{2}=-{L}_{c}$$

$b={b}_{w}+{b}_{b}={L}_{c}-1-{L}_{c}=-1,$ which demonstrates that ${b}_{b}$ can be much smaller than −1. q.e.d.
