# Supplementary material for: Influence of the mating design on the additive genetic variance in plant breeding populations
Source: Theor Appl Genet. 2023 Oct 31;136(11):236. doi: 10.1007/s00122-023-04447-2 (PMC10618341; doi:10.1007/s00122-023-04447-2)
Supplement: Supplementary file 1 — Supplementary file1 (PDF 1218 kb) [file 122_2023_4447_MOESM1_ESM.pdf]

# Influence of the mating design on the additive genetic variance in plant breeding populations

Tobias Lanzl, Albrecht E. Melchinger, Chris-Carolin Schön

Supplementary Table and Figures

***Table S1*** *Analysis of molecular variance (AMOVA) based on Euclidean distances between gametes from both ancestral populations Elite and Landrace. All variance components were significant (10,000 permutations, P < 0.05).*

| **Source of variation** | **Degrees of freedom** | **Variance component** |
|---|---|---|
| Between populations | 1 | 162.01 (35.1%) |
| Within populations | 228 | 299.46 (64.9%) |
| Within Elite | 114 | 281.12 (30.5%) |
| Within Landrace | 114 | 317.80 (34.4%) |
| Total | 229 | 461.44 |

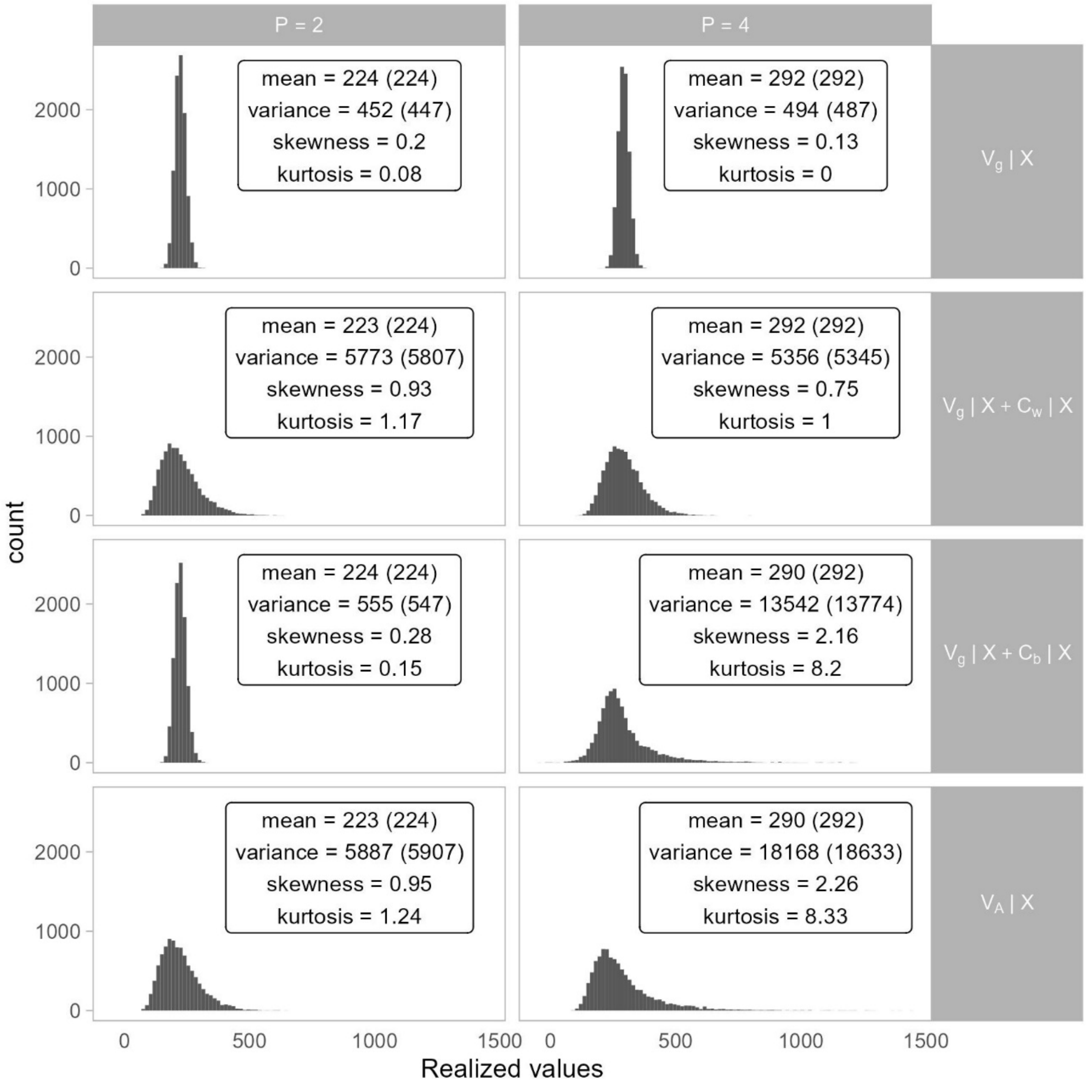


***Figure S1*** *Histograms of* $V_g|\mathbf{X}$, $V_g|\mathbf{X} + C_w|\mathbf{X}$, $V_g|\mathbf{X} + C_b|\mathbf{X}$, *and* $V_A|\mathbf{X}$ *for 10,000 samples of allele effects* $\mathbf{a} \sim N(\mathbf{0}, \mathbf{I})$ *for L = 1000 QTL using the disjoint cross (DC) mating design for* $P \in \{2,4\}$ *parental lines sampled from ancestral population Elite and N = 1000 genotypes for producing one replication of generation G1-DH. The values in the window give the mean, variance, skewness and kurtosis of the 10,000 realized values for each parameter. The values in the parentheses give the conditional mean and variance calculated for this replication and set of QTL using equations Eqs. 6, 7, 8, 9 and 10.*

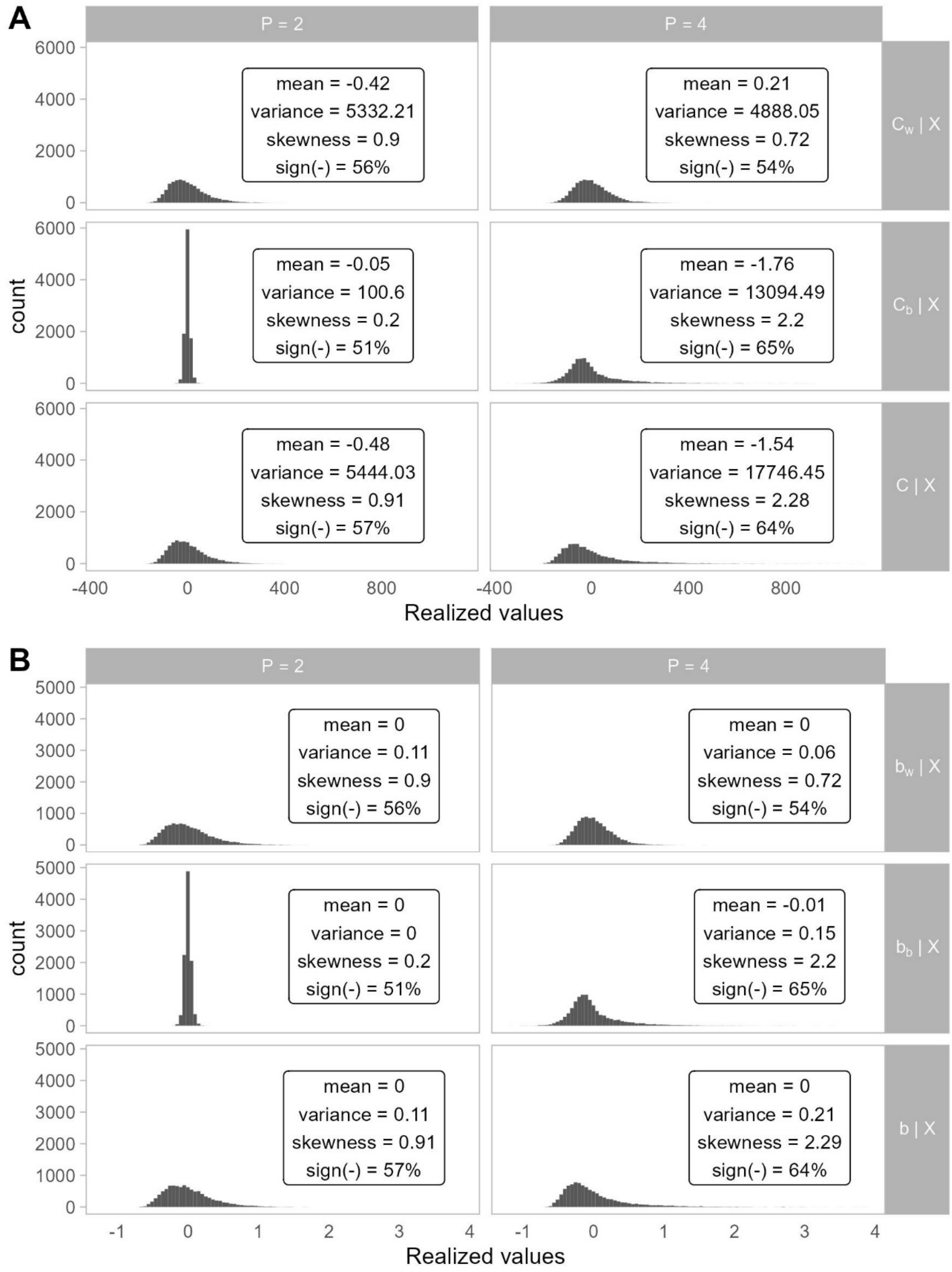


***Figure S2*** *Histograms of $C|\boldsymbol{X}$= $C_w|\boldsymbol{X} + C_b|\boldsymbol{X}$, $C_w|\boldsymbol{X}$ and $C_b|\boldsymbol{X}$ (A) and $b|\boldsymbol{X}$, $b_w|\boldsymbol{X}$, and $b_b|\boldsymbol{X}$ (B) for 10,000 samples of allele effects $\mathbf{a} \sim N(\mathbf{0}, \mathbf{I})$ for L = 1000 QTL using the disjoint cross (DC) mating design for $P \in \{2,4\}$ parental lines sampled once from ancestral population Elite and N = 1000 genotypes for producing one replication of generation G1-DH. The values in the window give the mean, variance, skewness and the percentage of values with a negative sign of the 10,000 realized values for each parameter.*

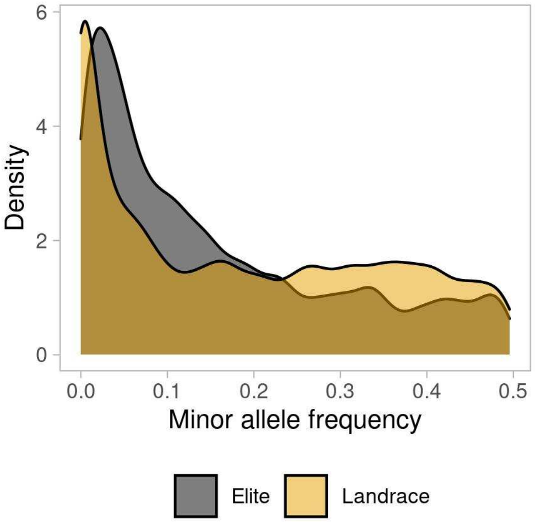


***Figure S3** Density estimation of the minor allele frequencies at the 2,500 potential QTL positions in ancestral population Elite (grey) and Landrace (yellow).*

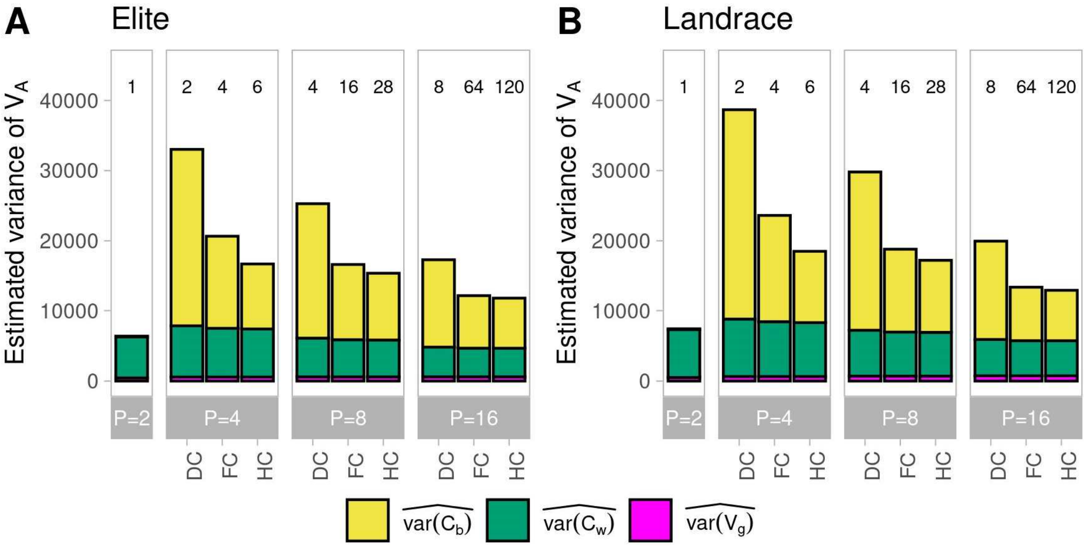


***Figure S4** Estimated variance of $V_A$ decomposed into the parts attributable to the genic variance ($\widehat{var}[V_g]$, magenta), the part attributable to covariances between QTL pairs on different chromosomes ($\widehat{var}[C_b]$, yellow) and on the same chromosome ($\widehat{var}[C_w]$, green) in generation G1-DH for different numbers of parental lines $P \in \{2,4,8,16\}$ sampled from ancestral population Elite (A) and Landrace (B) and using three mating designs (disjoint cross (DC), factorial cross (FC), and half-diallel cross (HC)) for producing generation G1 and N = 1000 genotypes for producing G1-DH and L = 1000 QTL. The number of crosses generated in the respective mating design is shown above the bars.*

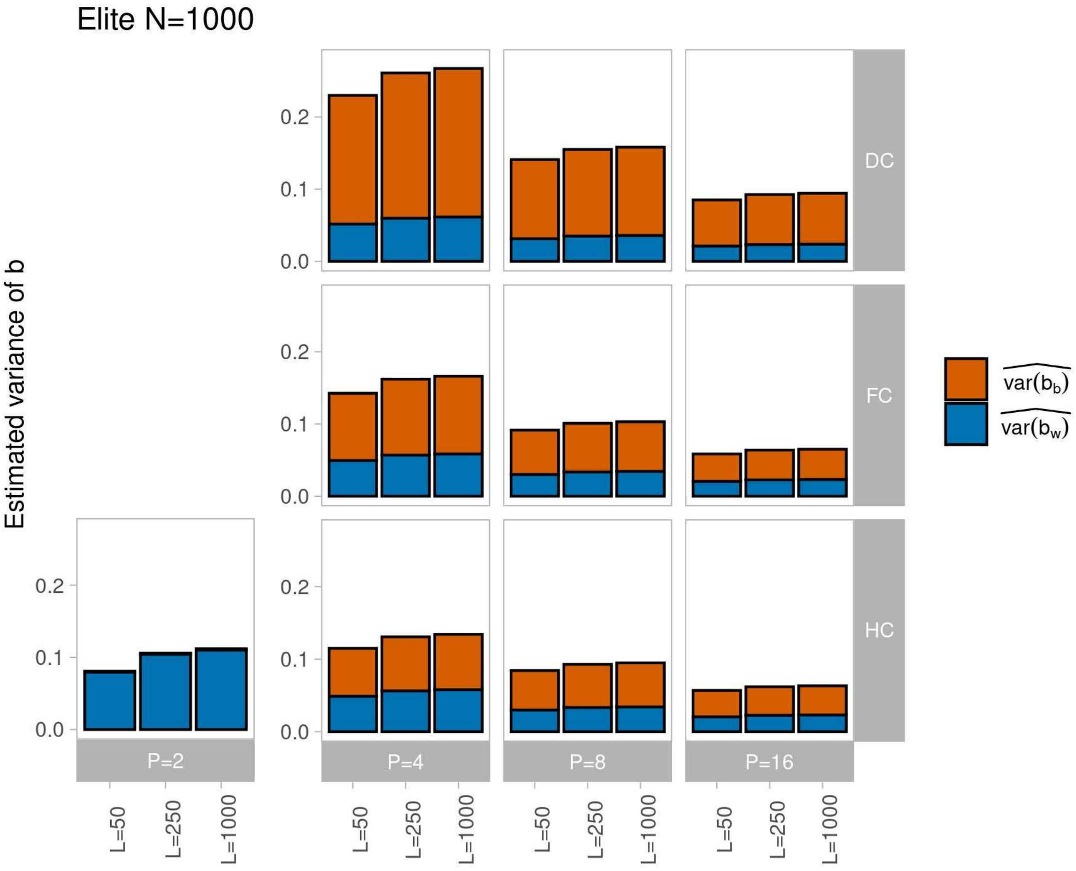


***Figure S5*** *Estimated variance of b decomposed into the parts attributable to QTL pairs on different chromosomes ($\widehat{var[b_b]}$, red) and on the same chromosome ($\widehat{var[b_w]}$, blue) in generation G1-DH for different numbers of parental lines $P \in \{2,4,8,16\}$ sampled from ancestral population Elite using three mating designs (disjoint cross (DC), factorial cross (FC), and half-diallel cross (HC)) for producing generation G1 in scenarios with N = 1000 genotypes and varying numbers of QTL $L \in \{50, 250, 1000\}$.*

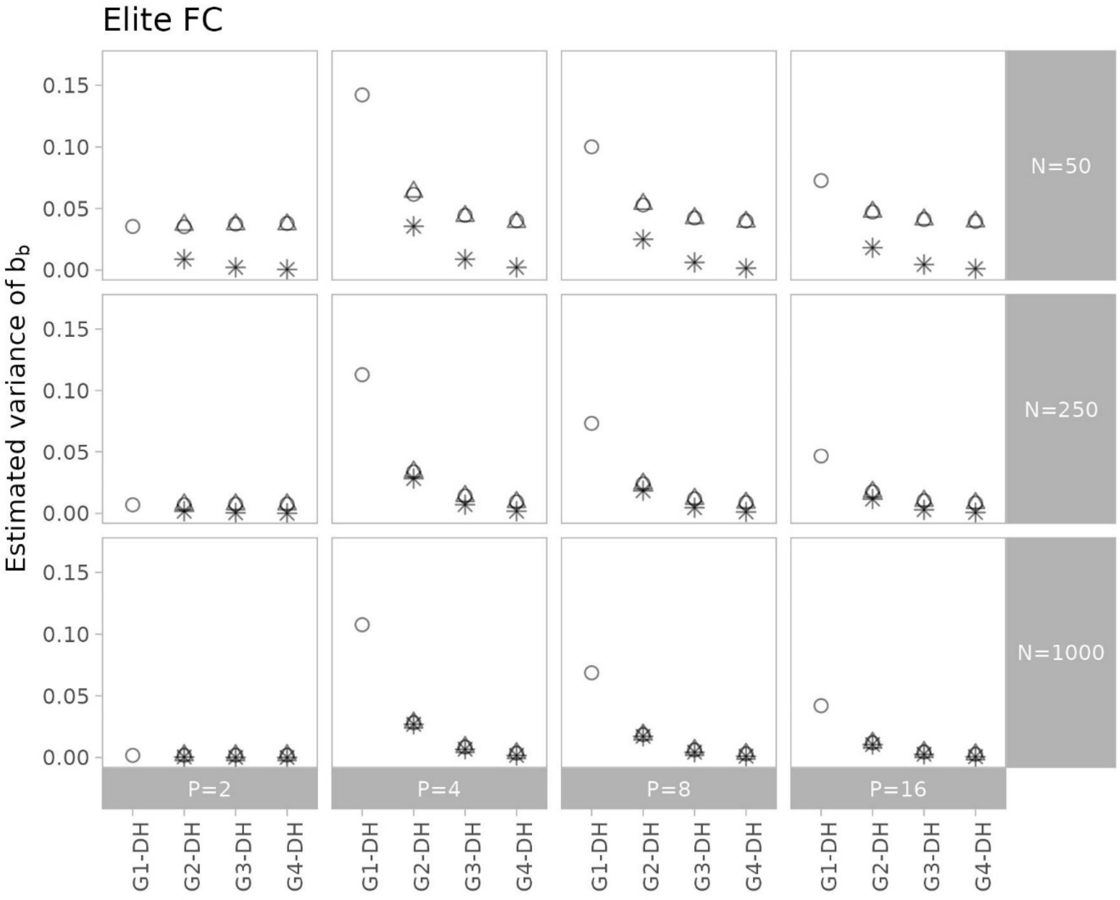


***Figure S6*** *Decay of the estimated variance of $b_b$ from generation G1-DH to G4-DH for varying numbers of parental lines $P \in \{2, 4, 8, 16\}$ sampled from ancestral population Elite using the mating design factorial cross (FC) with $N \in \{50, 250, 1000\}$ genotypes for producing generations G1 to G4 and G1-DH to G4_DH with L = 1000 QTL. The circles show the variance of $b_b$ estimated in the simulations, the asterisks show the expected decay based on the value in G1-DH for a population with $N = \infty$ and the triangles show the decay estimated with the non-linear regression with Eq. 19.*

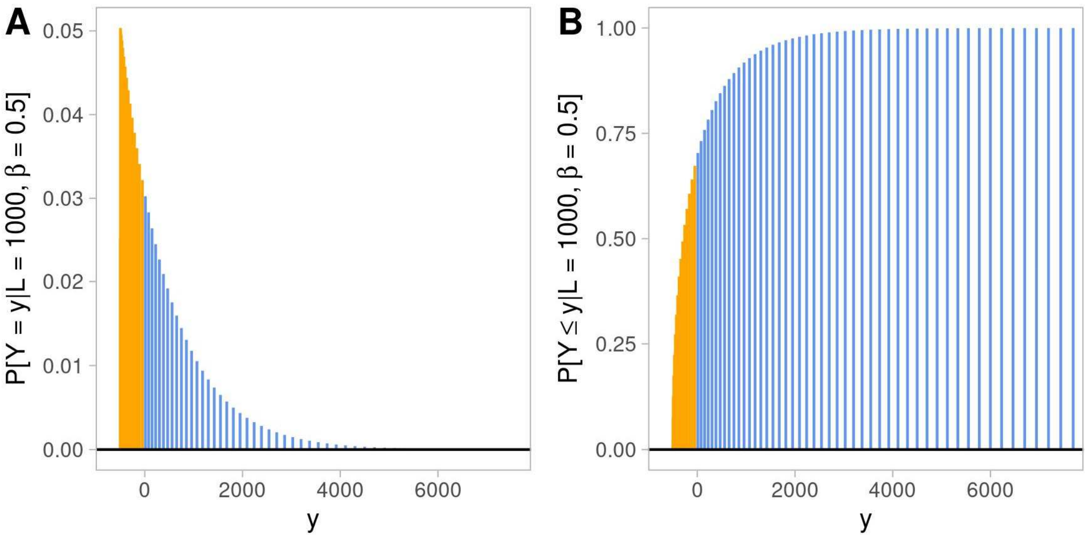


***Figure S7*** *Probability mass function of P[Y=y ;* $L = 1000, \beta = 0.5$*] (A) and cumulative distribution function of* $P[Y \leq y; L = 1000, \beta = 0.5]$ *(B), where Y is the random variable referring to the difference in the number of QTL pairs, where the product of the allele effects is positive, minus the number of QTL pairs, where it is negative, for L = 1000 QTL assuming that the probability of a positive effect of the reference allele is* $\beta = 0.5$*. The events with* $Y < 0$ *and* $Y \geq 0$ *are colored in orange and blue, respectively.*

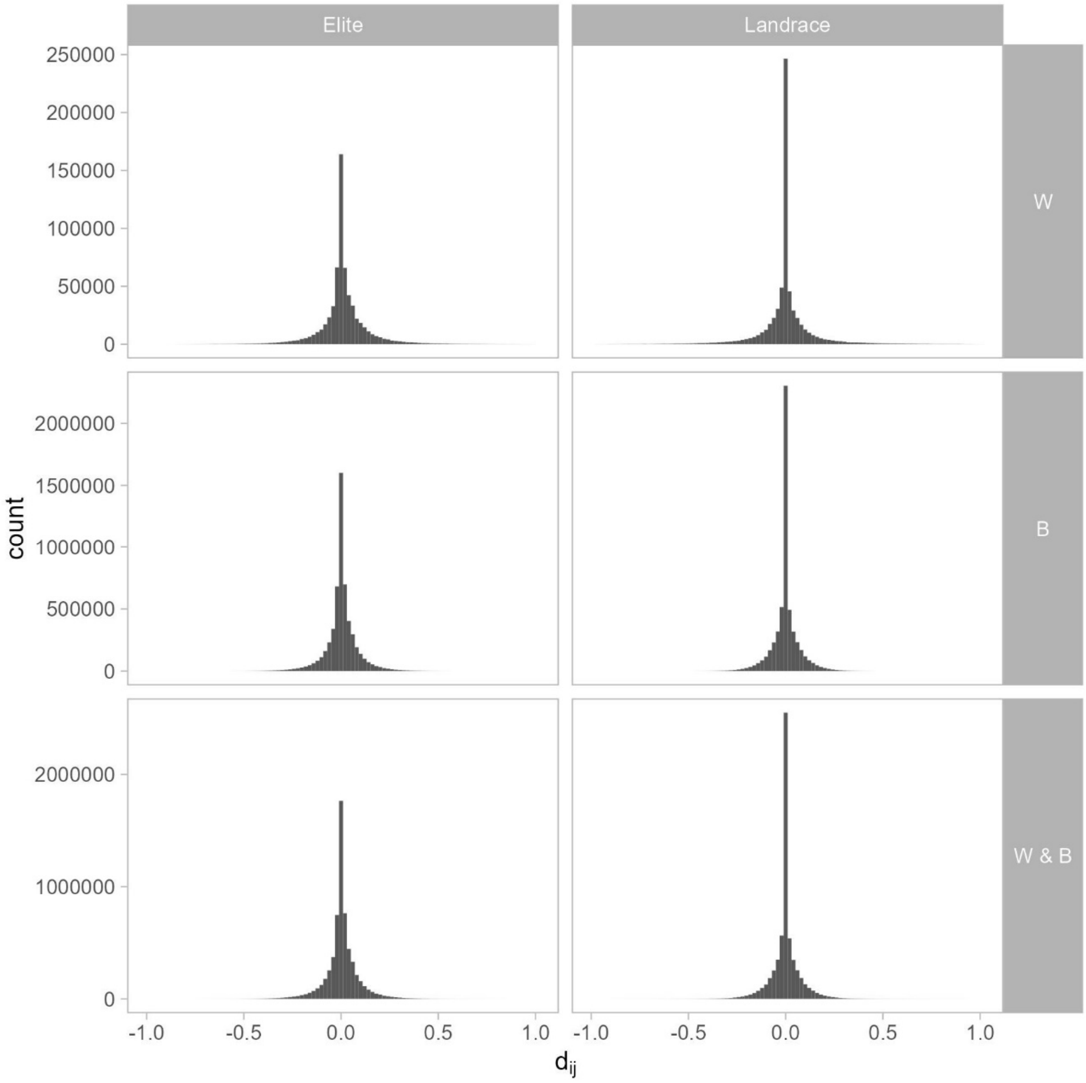


***Figure S8*** *Histograms for the off-diagonal elements* $d_{ij}$ *(4 x gamete phase disequilibrium) of matrix* **D** *for pairs of 2500 QTL located on the same (***W***) or on different chromosomes (***B***), as well as for all elements in* **W** *&* **B** *for ancestral populations Elite and Landrace.*
